# Single-cell RNA-seq and pathological phenotype reveal the functional atlas and precise roles of Sox30 in testicular cell development and differentiation

**DOI:** 10.1038/s41419-025-07442-1

**Published:** 2025-02-19

**Authors:** Cheng-ting Xie, Hui-lian Zhang, Yi Li, Qian Li, Yi-xian Wen, Jin-yi Liu, Fei Han

**Affiliations:** 1 School of Public Health, Chongqing Medical University, Chongqing, 400016 China; 2 Joint International Research Laboratory of Reproduction and Development of the Ministry of Education, Chongqing, 400016 China; 3 Institute of Toxicology, College of Preventive Medicine, Army Medical University, Chongqing, 400038 China

**Keywords:** Differentiation, Meiosis, Infertility

## Abstract

Sox30 has recently been demonstrated to be a key regulator of spermatogenesis. However, the precise roles of Sox30 in the testis remain largely unclear. Here, the specific functions of Sox30 in testicular cells were determined by single-cell sequencing and confirmed via pathological analyses. Sox30 loss appears to damage all testicular cells to different extents. Sox30 chiefly drives the differentiation of primary spermatocytes. Sox30 deficiency causes spermatocyte arrest at the early phase of meiosis I, with nearly no normally developing second spermatocytes and three new spermatocyte -subclusters emerging. In addition, Sox30 seems to play important roles in the mature phenotypes of Sertoli and Leydig cells, and the proliferation and differentiation of spermatogonia. The developmental trajectory of germ cells begins with spermatogonia and splits into two different spermatocyte branches, with Sox30-null spermatocytes and wild-type spermatocytes placed at divergent ends. An opposite developmental trajectory of spermatocyte subclusters is observed, followed by incomplete development of spermatid subclusters in Sox30-null mice. Sox30 deficiency clearly alters the intercellular cross-talk of major testicular cells and dysregulates the transcription factor networks primarily involved in cell proliferation and differentiation. Mechanistically, Sox30 appears to have similar terminal functions that are involved mainly in spermatogenic development and differentiation among major testicular cells, and Sox30 performs these similar crucial roles through preferential regulation of different signalling pathways. Our study describes the exact functions of Sox30 in testicular cell development and differentiation and highlights the primary roles of Sox30 in the early meiotic phase of germ cells.

## Introduction

Reproduction is a vital characteristic of life, because any form of life can occur and continue in various modes via reproduction [1, 2]. To achieve fertility, a male requires a set of perfect and wholesome capabilities for sperm production [3], which primarily depend on the normal development of both testicular germ cells and somatic cells, and the maintenance of fertility hinges on the continuous production of sperm [4, 5]. In mammals, the testis develops *in utero* when the Y chromosome gene sex-determining region Y (SRY) is expressed. SRY initiates a genetic program, involving Sox9 that directs a subset of bipotential progenitor cells differentiate into Sertoli cells, which orchestrate further testis development [4, 6, 7]. Testicular somatic cells contribute to morphogenesis of the foetal testis, and the dynamic regulation of different types of testicular somatic cells provides a highly specialized microenvironment, the testis niche, to support the normal development and differentiation of germ cells [5]. Substained-steady spermatogenesis is driven by the balance between the self-renewal and differentiation of spermatogonial stem cells (SSCs), alongside highly complex germ cell–niche interactions [8–10]. On the basis of the balance of their self-renewal and differentiation, SSCs then undergo niche-guided transitions between multiple cell states and cellular processes, including a commitment to mitosis, meiosis, and the subsequent stage of sperm maturation accompanied by chromatin repackaging and morphological changes [8, 11, 12]. Although considerable progress in considering the biological process of gametogenesis and germline-niche communication has been achieved [5, 8, 9, 13], the finely programmed and developmental molecular events of gametogenesis remain largely unknown.

SRY-box (Sox) proteins are a family of transcription factors that contain a highly conserved high mobility group (HMG)-box for DNA-binding [7, 14]. The mammalian Sox family is composed of approximately 20 members, including the testis-determining factors Sry and Sox9, and is widely involved in diverse developmental processes and tissue systems, including preimplantation development, endoderm induction, cell fate commitment and reprogramming, haematopoiesis, and sex determination and differentiation [7, 14–16]. Sox factors may be closely associated with developmental disorders and various diseases, including cancer. An increasing number of studies have confirmed the potential of Sox family members in the diagnosis and treatment of developmental diseases and tumours [17, 18] Among Sox family members, Sox30, the only member of subfamily H, is a relatively unkonwn member. In recent years, SOX30 has been shown to play important roles in tumorigenesis, including in lung cancer, ovarian cancer, and other types of cancer [19–22]. However, although Sox30 is a male-specific gene that is highly expressed in adult testes but not in ovaries [23, 24], the major roles of Sox30 in male development and reproduction seem to have been ignored for a long time.

On the basis of the positive expression pattern of Sox30 in testicular development, Sox30 has been proposed to be involved in spermatogenic differentiation and spermatogenesis in mice [24–26]. Studies have demonstrated that Sox30 is a key regulator of spermiogenesis [27]. Sox30-knockout mice exhibit specific testicular pathological defects with lower testicular size and weight concomitant with sterility without spermatozoa, whereas fertile spermatozoa are observed after Sox30 is re-expression in the knockout mice [27–29]. Although the importance of Sox30 in regulating spermatogenesis has gradually become understood, the specific stage at which Sox30 predominantly functions in spermatogenic cells is still controversial. Sox30 is considered to have a role work in later meiotic cells and postmeiotic haploids, as spermiogenesis is arrested at the early round spermatid stage in mutant males [27, 30]. Mechanistically, Sox30 controls the transition from a late meiotic to a postmeiotic gene expression program and subsequent round spermatid development [31]. However, the rich expression of Sox30 seems to be restricted meiotic spermatocytes and round spermatids, and Sox30 expression is clearly greater in meiotic spermatocytes than in round spermatids [29, 31, 32], suggesting that Sox30 may act before round spermatid formation. Our previous study revealed that the germ cells of Sox30-null male mice are arrested during the meiotic process, markedly impaired in the zygotene-to-pachytene transition, and concomitant abnormal proliferation of Leydig cells [29]. Mechanistically, Sox30 directly targets the meiotic genes *Stra8*, *Rec8* and *Cyp26b1*, which are involved in the retinoic acid (RA) signalling pathway, and it directly or indirectly regulates a group of sex differentiation genes (including *Sox9, Wnt4*, *Rspo1* and *Ctnnb1*) [29]. These data imply that Sox30 likely plays a role in germ cell differentiation and even sex determination in early developmental stages. However, the precise roles of Sox30 in testicular cell development and differentiation remain largely unexplored.

In this study, we conducted single-cell RNA sequencing on testicular tissue from a successfully constructed Sox30-null mouse model. Combined with transcriptional profiles of testicular cells, 10 testicular cell clusters and their proportional change were identified in Sox30-null mice. The major results of the cell proportional changes were further confirmed by pathological phenotype analyses. The subclusters of the major testicular cells and their proportional variation were further analysed in Sox30-null mice. Loss of *Sox30* resulted in spermatocytes arrest at meiosis I, suggesting that Sox30 primarily drives the development and differentiation of spermatocytes. Moreover, the aberrant germ cell differentiation of Sox30-null mice likely begins as early as spermatogonial process, and Sox30 plays a role in regulating the mature phenotypes of Sertoli cells and Leydig cells. The developmental pseudotime trajectory of testicular cells was subsequently constructed, which revealed that the development of spermatocytes was influenced mainly by Sox30. In addition, intercellular communication and transcription factor regulatory network analyses were performed, and revealed that Sox30 plays essential roles in the cross-talk of testicular cells and maintaining the spermatogenic microenvironment. Our present study preliminarily presents the functional features of Sox30, and reveals the specific roles of Sox30, in testicular cell development and differentiation.

## Materials and methods

### Generating Sox30-null mice

Sox30-null mice were generated by the Model Animal Research Center at Nanjing University, and the construction strategy has been described in our previously reports [29, 33]. Briefly, the targeted gene including homologous arms was retrieved from a BAC vector, and a LoxP-SA-IRES-GFP-NEO-STOP-PPS-LoxP cassette was inserted between Exon1 and Exon2 of Sox30 via homologous recombination. The targeting vector was validated by PCR, enzyme digestion, and sequencing, then linearized with AsiSI and electroporated into C57BL/6 embryonic stem (ES) cells. Recombinants were selected under G418 and ganciclovir (Ganc). Targeted ES cells were screened by PCR and Southern blot, and positive clones were chosen for microinjection to generate chimeras. The chimeras were crossed with C57BL/6 mice to produce heterozygous offspring. The mice were maintained in a specific pathogen-free (SPF) facility under a 12-h light/12-h dark cycle with ad libitum access to water and food. The genotype of the offspring was identified by PCR. All mouse experiments were conducted with the approval of the Institutional Animal Care and Use Committee of Chongqing Medical University and Army Medical University, China.

### Hematoxylin-eosin staining analysis

Testes were dissected from mice of different genotypes and immediately fixed in Bouin’s fluid (Scientific Phygene Inc, China). The fixed testes were dehydrated, embedded in paraffin, and sectioned into 5 μm thick slices. These sections were de-waxed, rehydrated, and stained with hematoxylin and eosin (H&E; Beyotime, Shanghai, China).

### Cell proliferation analysis

The proliferation of testicular cells was assessed using 5-ethynyl-2’-deoxyuridine (EdU). Mice of different genotypes were intraperitoneally injected with 100 µg of EdU. Seventy-two hours after injection, the proliferation assay of testicular cells was performed using an EdU detection kit (Ribobio, Guangzhou, China) according to the manufacturer’s instructions.

### Immunofluorescence analysis

Testis tissues from mice of different genotypes were harvested into ice-cold PBS, fixed with freshly prepared 4% paraformaldehyde for 30 min, and punctured with a needle. After overnight fixation, the tissues were incubated in 15% sucrose for one day and then in 30% sucrose for another day. The tissues were frozen in liquid-cooled isopentane. Sections (5 µm thick) were obtained and placed onto Fisherbrand Colorfrost Plus slides, then air-dried. The section slides were washed with PBS and permeabilized with 0.1% Triton X-100 for 1 h. These sections were quenched with 0.1 M glycine, washed with PBS, and blocked with 10% foetal bovine serum. They were then incubated with the primary antibody, Sycp3(Scp3) mouse monoclonal antibody (1:100, sc-74569, Santa Cruz Biotechnology), followed by the fluorescent secondary antibody, DyLight 488 Goat Anti-Mouse IgG (1:100, A23210, Abbkine). The slides were observed under a fluorescence microscope (Super-Resolution Mocroscope system, C2/SIME, Nikon, Japan).

### Immunohistochemistry (IHC) analysis

Paraffin-embedded testis tissue sections (5 µm thick) were baked at 60 °C for 2 h, deparaffinized in xylene, and rehydrated through graded alcohol series to water. The sections were immersed in citrate buffer and subjected to antigen retrieval for 15 min at 95 °C. The tissue sections were then blocked with 0.3% H_2_O_2_ and, treated with 10% normal goat serum for 15 min. They were incubated with the primary antibody, Cyp26b1 (1:100, 21555-1-AP, proteintech) overnight at 4 °C, followed by incubation with a biotinylated secondary antibody (1:2000, Abbkine) for 2 h at room temperature. The slides were sealed with neutral resin (Solarbio, Beijing, China), and photographs were taken under a light microscope (Olympus, Japan).

### Single-cell solution preparation

Fresh testicular tissues were removed from sacrificed and dissected mice of different genotypes randomly, and immediately stored in MACS Tissue Storage Solution (Miltenyi Biotec, Germany) on ice. To generate sufficient material, testicular tissues from three 10-week-old mice of the same genotype were pooled. The testicular tissues were cut into about 1 mm^3^ pieces, washed in PBS, and digested using the Solo^TM^ Dissociation Kit (Sinotech Genomics, JZ-SC-58201) for 45 min at 37 °C. The enzymatic digestion was stopped by adding excess RPMI-1640 medium, and the cell solution was filtered through a 40 μm cell strainer. The cells were then sorted using a MACS Dead Cell Removal Kit (Miltenyi Biotec) to remove dead cells. Living cells were resuspended, and the prepared single cell suspension was kept on ice until loaded onto the Chromium controller instrument (10× Genomics) for single-cell transcriptome.

### Single-cell transcriptome capturation, library construction and sequencing

The prepared cells were loaded into Chromium microfluidic chips and barcoded using a 10× Chromium Controller (10× Genomics). Single-cell transcriptomes were reverse-transcribed into cDNA libraries containing 10× cell barcodes and unique molecular identifiers (UMIs). All procedures were performed using reagents from the Chromium Next GEM Single Cell 5’ Library & Gel Bead Kit v1.1 (10× Genomics, Cat. No. 1000165) according to the manufacturers’ protocol. The libraries were sequenced in PE150 mode (pair-end for 150 bp reads) on the NovaSeq platform (Illumina). Raw sequencing reads from the cDNA libraries were mapped to the reference genome using 10× Genomics Cell Ranger (v3.1.0).

### Sequencing data processing

Raw sequencing reads of the cDNA library were mapped to the reference genome using 10× Genomics Cell Ranger (v3.1.0). Genome Reference Consortium Mouse Build 38 (GRCm38) was used as the reference genome for the 10× Cell Ranger.

### Contamination removal of single-cell sequencing

When visualizing the expression levels of specific cell marker genes using FeaturePlot in the Seurat package v3.1.2, ambient RNA contamination was detected. To address this issue, ambient RNA removal was performed using the decontx algorithm in the celda package v.1.3.8 [34]. The decontx algorithm assumes the presence of K cell populations and employs Bayesian variational inference to estimate the ambient RNA contamination as a weighted combination of the specific cell population distributions. This algorithm requires the raw UMI counts and several cell populations, identified using the Find Clusters function in the Seurat package v3.1.2 as input. As a result, a decontaminated count matrix was derived from the raw data. A default random seed was used throughout the analyses to ensure reproducibility.

### Quality control, dimension-reduction and clustering of single-cell sequencing

Cells were filtered based on gene counts ranging from 200 to 5,000 and UMI counts below 30,000. Cells with more than over 20% mitochondrial content were removed. The Seurat v3.1.2 package [35] was used for dimensionality reduction and clustering. Gene expression data were normalized and scaled using the Normalize Data and Scale Data functions, respectively. The top 2000 variable genes identified using the Find Variable Features function and were selected for principal component analysis (PCA). Based on the top 20 principle components, cells were clustered using the Find Clusters function. Batch effect between samples were corrected using Harnomy [36]. Finally, uniform manifold approximation and projection (UMAP) algorithm was applied to visualize cells in a two-dimensional space.

### Cell type annotation of single-cell sequencing

The cell type for each cluster was identified based on the expression of canonical markers found among the DEGs using the SynEcoSys database. Heatmaps and dot plots displaying the expression of these markers were generated using the DoHeatmap, DotPlot, and Vlnplot functions in Seurat v3.1.2.

### Differentially expressed genes (DEGs) analysis of single-cell sequencing

To identify differentially expressed genes (DEGs), the Find Markers function in Seurat, based on the Wilcox likelihood-ratio test with default parameters, was used. Genes were selected as DEGs if they were expressed in more than 10% of the cells within a cluster and had an average log fold change (FC) value greater than 0.25 or less than -0.25. For the annotation of cell types in each cluster, the expression of canonical markers found among the DEGs was evaluated using literature knowledge. The expression of markers for each cell type was visualized using heatmaps, dot plots, and violin plots generated with the DoHeatmap, DotPlot, and Vlnplot functions in Seurat. Cells identified as doublet, expressing markers for different cell types, were manually removed.

### Function and pathway enrichment analysis of single-cell sequencing

To investigate the potential functions and pathways of DEGs, the Gene Ontology (GO) and Kyoto Encyclopedia of Genes and Genomes (KEGG) analyses were performed using the “clusterProfiler” R package (version 3.16.1). Functions and pathways with an adjusted p-value(p-adj) less than 0.05 were considered significantly enriched. GO gene sets including molecular function (MF), biological process (BP), and cellular component (CC) categories were used as references.

### Pseudotime trajectory analysis of single-cell sequencing

Cell differentiation trajectories were reconstructed using Monocle2 [37]. DEGs were utilized to sort cells in according to their spatiotemporal differentiation order. Dimensionality reduction was performed using the DDRTree method after identifying variable features with the Find Variable Features function. The differentiation trajectory was visualized using the plot_cell_trajectory function. To predict the differentiation potential of monocyte subpopulations, the CytoTRACE method was employed. CytoTRACE is a computational approach that estimates the differentiation state of cells from single-cell RNA-sequencing data using gene counts and expression levels [38].

### Cell-cell interaction analysis of single-cell sequencing

Cell-cell interaction analyses were performed using CellPhoneDB v2.1.0 [39] based on known receptor-ligand interactions between cell types/subtypes. Cluster labels of all cells were randomly permuted for 1000 times to calculate the null distribution of average ligand-receptor expression levels for the interacting clusters. The expression threshold for individual ligands or receptors was set based on the average log gene expression distribution across all the cell types. Significant cell-cell interactions were defined as those with *p* value less than 0.05 and an average log expression greater than 0.1, and were visualized using the circlize v0.4.10 R package.

CellCall v0.0.0.9000 [40] was used to analyse intercellular interactions based on receptor-ligand interactions between cell types/subtypes and to infer the signalling pathways involved in internal regulation. The fraction of ligand-receptor genes interactions between cell types/subtypes was assessed by integrating the L2 norm of the ligand-receptor interaction and the activity fraction of downstream TFs, calculated using the built-in GSEA algorithm. Ligand-receptor-TF interaction with significant interactions between cell types/subtypes were selected using a hypergeometric test with a *p* value of less than 0.05. Visualization was performed by the built-in plotting functions from CellCall.

### Transcription factor regulatory network analysis of single-cell sequencing

The transcription factor network was constructed using pyscenic v0.11.0 [41] with the scRNA-seq expression matrix and transcription factors from AnimalTFDB. GRNBoost2 was used to predict a regulatory network based on the co-expression of regulators and their targets. CisTarget was then applied to filter out indirect targets and to identify transcription factor binding motifs. Subsequently, AUCell was used to quantify regulon activity for every cell. Top TF regulons with high Regulon Specificity Score (RSS) were visualized using the heatmap function in R.

### Statistical analysis

Data are presented as mean ± standard error of mean (SEM). Differences were compared using two-tailed unpaired *t*-tests or non-parametric test, and *p* values were calculated using GraphPad prism 8.0 (Graph-Pad Software Inc., USA). A *p* value less than 0.05 was considered statically significant.

## Results

### The altered landscape of testicular cell populations in Sox30-null mice

**Fig. 1:**
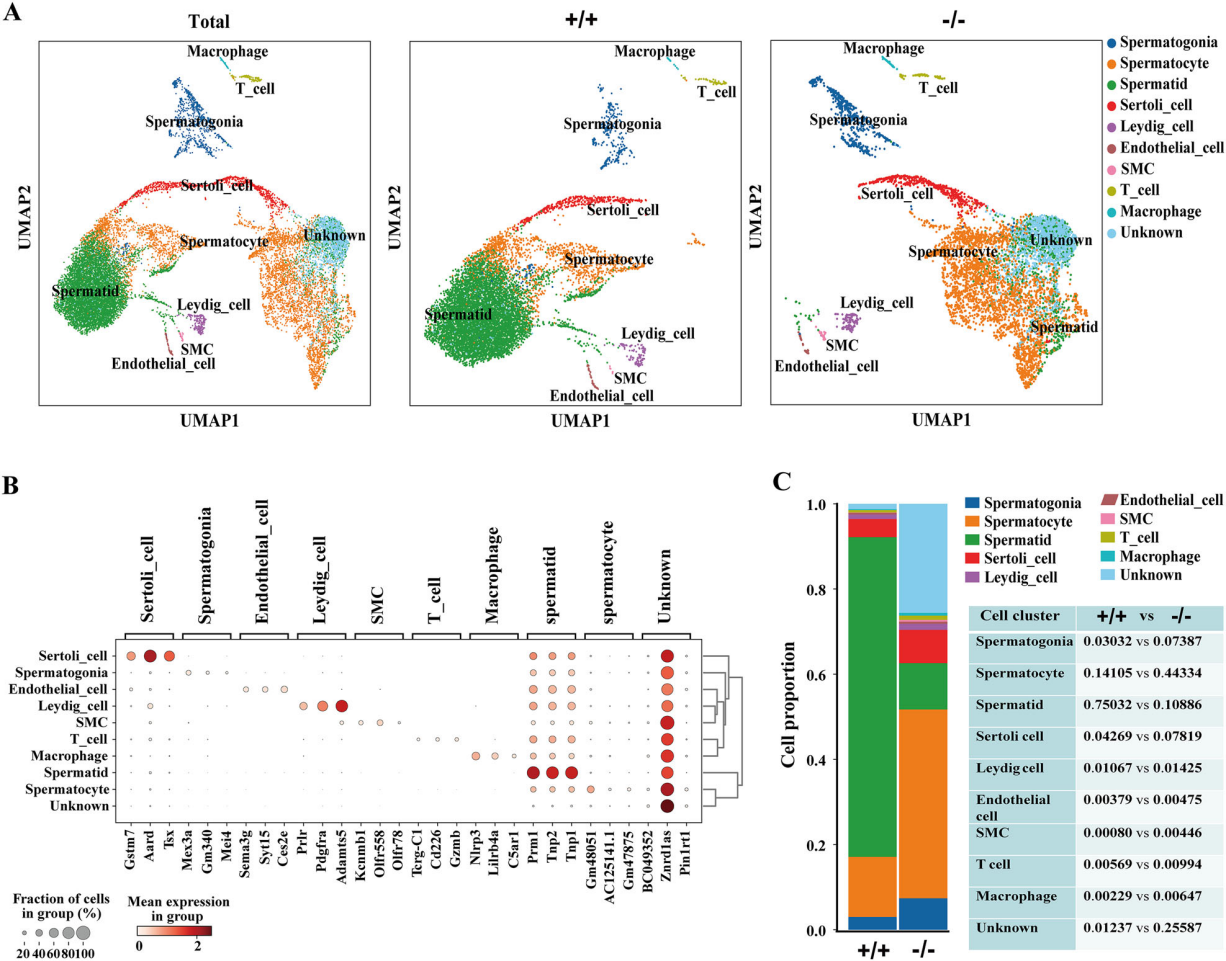
Overview of major cell types, cellular attributes and cell proportions inferred from scRNA-seq in Sox30^+/+^ and Sox30^−/−^ mouse testes. **A** UMAP plot showing the heterogeneity of cell clusters the integrated (Total), Sox30^+/+^ (+/+) and Sox30^−/−^ (−/−) datasets. Ten clusters were identified based on the expression of marker genes, with each colour representing a different cell cluster. **B** Bubble chart displaying the expression of 30 representative marker genes for the ten testicular cell clusters. **C** Bar graphs (left) and a table (right) illustrating the proportions of different testicular cell clusters in Sox30^+/+^ (+/+) and Sox30^−/−^ (−/−) mice.

To characterize the baseline cellular diversity and differences in the cell types of testes from mice with different genotypes of Sox30, we profiled testicular cells from wild-type (Sox30^+/+^) and Sox30-null (Sox30^−/−^) mice via single-cell sequencing. A total of ten cell clusters were identified in the whole-cell population on the basis of the expression of known cell type-specific markers (Fig. 1A, B, Supplementary Table S1). The known prototypical markers of Sertoli cells (e.g., *Gstm7*, *Aard*, *Tsx*, *Wt1* and *Vim*), endotheliocytes (e.g., *Sema3g*, *Syt15*, *Ces2e*, *Cdh5* and *Vwf*), Leydig cells (e.g., *Prlr*, *Pdgfra*, *Adamts5*, *Star* and *Fabp3*), smooth muscle cells (SMCs; e.g., *Kcnmb1*, *Olfr558*, *Olfr78*, *Acta2* and *Myh11*), T cells (e.g., *Tcrg-C1*, *Cd226*, *Gzmb*, *Ptprc* and *Cd3g*) and macrophages (e.g., *Nlrp3*, *Lilrb4a*, *C5arl*, *Apoe* and *Csf1r*) were found in the somatic cell clusters. The known markers distinguishing spermatogonia (e.g., *Mex3a*, *Gm340*, *Mei4*, *Uchl1* and *Stra8*), spermatocytes (e.g., *Gm48051*, *AC12514.1*, *Gm47875*, *Rad51ap2* and *Cdc42ep3*) and spermatids (e.g., *Prm1*, *Tnp1*, *Tnp2*, *Spata9* and *Lyzl6*) were observed in the germ cell clusters. Notably, the known genes encoding markers of spermatocytes (such as *Znrd1as* and *Malsu1*) and round spermatids (such as *BC049352* and *Ccdc81*) were highly expressed in the unknown cell cluster, suggesting that this cell cluster may possess some characteristics of both spermatocytes and spermatids.

To gain insight into the cellular differences between testes from Sox30^+/+^ and Sox30^−/−^ mice, we analysed the proportion of each testicular cell type in Sox30^+/+^ and Sox30^−/−^ mice (Fig. 1C). Compared with those in Sox30^+/+^ mice, the proportions of spermatogonia (0.03032 in Sox30^+/+^ mice vs. 0.07387 in Sox30^−/−^ mice) and spermatocytes (0.14105 in Sox30^+/+^ mice vs. 0.44334 in Sox30^−/−^ mice) in Sox30^−/−^ mice were increased more than two and three times in quantity, respectively (Fig. 1C). Conversely, the proportion of spermatids (0.75032 in Sox30^+/+^ mice vs. 0.10886 in Sox30^−/−^ mice) was decreased more than sixfold in Sox30^−/−^ mice (Fig. 1C). The proportion of SMCs (0.00080 in Sox30^+/+^ mice vs. 0.00475 in Sox30^−/−^ mice) was increased more than fivefold in Sox30^−/−^ individuals, and the proportions of macrophages (0.00229 in Sox30^+/+^ mice vs. 0.00647 in Sox30^−/−^ mice), T cells (0.00569 in Sox30^+/+^ mice vs. 0.00994 in Sox30^−/−^ mice), Sertoli cells (0.04269 in Sox30^+/+^ mice vs. 0.07919 in Sox30^−/−^ mice), and Leydig cells (0.01067 in Sox30^+/+^ mice vs. 0.01425 in Sox30^−/−^ mice) were also increased in Sox30^−/−^ mice (Fig. 1C). However, little change in the proportion of endothelial cells (0.00379 in Sox30^+/+^ mice vs. 0.00475 in Sox30^−/−^ mice) was observed in Sox30^−/−^ mice (Fig. 1C). Notably, the proportion of the unknown cluster (0.01237 in Sox30^+/+^ mice vs. 0.25587 in Sox30^−/−^ mice) was increased approximately twentyfold in Sox30^−/−^ mice (Fig. 1C). Given the variation in the quantity of the unknown cluster and the fact that it contains characteristics of both spermatocytes and spermatids, we speculate that this cell type is probably the multi-nucleated cells that produced in Sox30^−/−^ mice, as reported previously [29].

The single-cell data of testis tissues clearly indicated that Sox30-null strongly impacts the unknown, spermatid, spermatocyte, and SMC clusters, and the differences of these cells were all greater than three times in quantity in Sox30^−/−^ mice than in Sox30^+/+^ mice. According to the quantitative data for testicular cells, the most affected cell type associated with Sox30 loss was the multi-nucleated cells (the unknown cells), followed by spermatids, SMCs, spermatocytes, macrophages, spermatogonia, Sertoli cells, T cells, Leydig cells, and endothelial cells.

### The precise effects of Sox30 on testicular subcell type populations

**Fig. 2:**
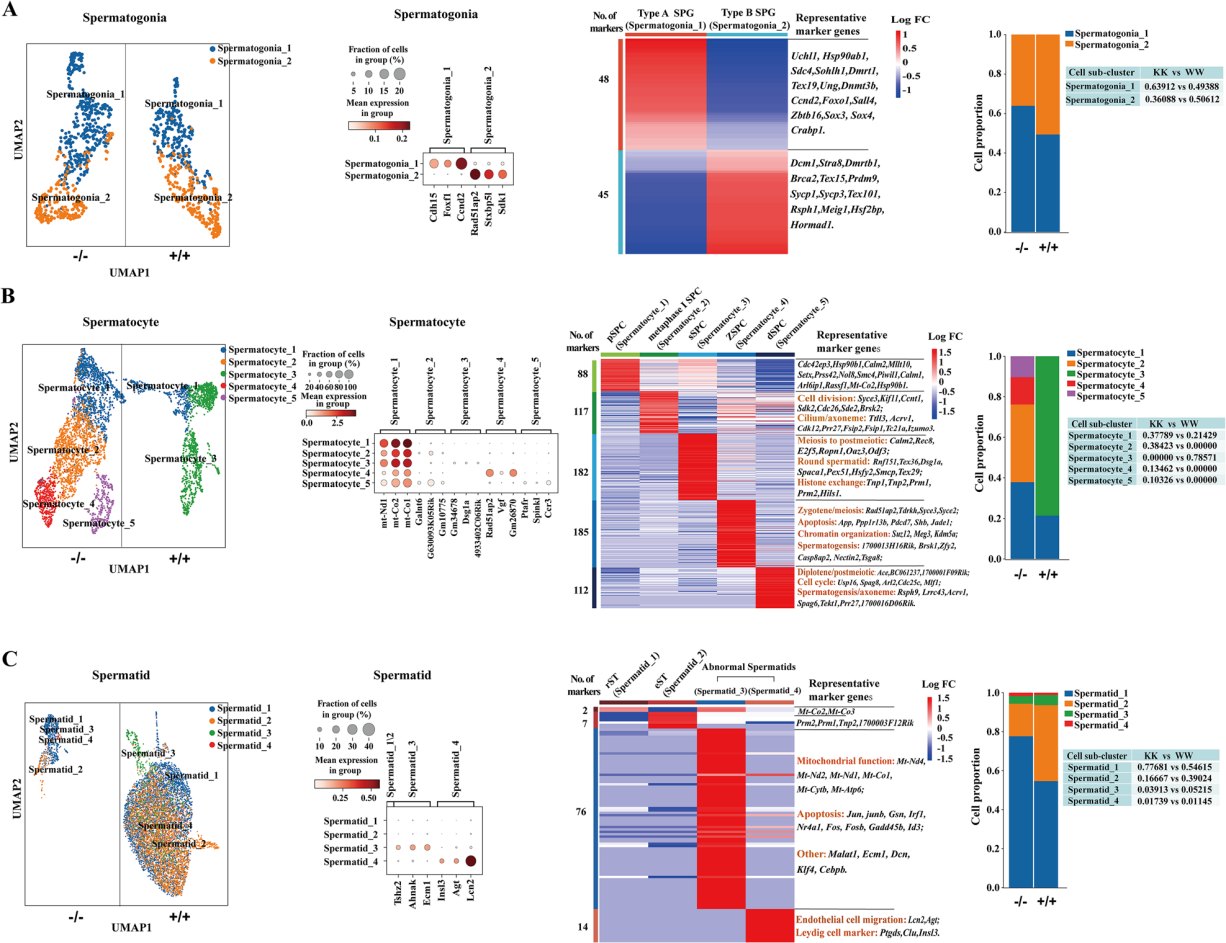
Identification and cell proportion of sub-clusters in major testicular cells. From left to right: UMAP plot of the spermatogonia (**A**), spermatocytes (**B**), and spermatids (**C**) datasets from Sox30^+/+^ (+/+) and Sox30^−/−^ (−/−) mice. Each sub-cluster was identified based on the expression of marker genes. Bubble chart showing the expression of three representative marker genes for each subcluster. Heatmaps displaying the expression of representative marker genes of spermatogonia (**A**), spermatocytes (**B**) and spermatids (**C**) from each sub-cell cluster. Bar graphs (left) and tables (right) represent the cell proportions of different sub-cell clusters in Sox30^+/+^ (+/+) and Sox30^−/−^ (−/−) mice.

To further determine the precise effects of Sox30 on testicular cells, we subclustered the major cell types, including the following germ cell clusters: spermatogonia, spermatocytes and spermatids, and the following somatic cell clusters: Sertoli cells and Leydig cells. Given the limited number of remaining cell clusters, subcluster analysis was not performed. Among the germ cell clusters, two subclusters of spermatogonia, five subclusters of spermatocytes, and four subclusters of spermatids were observed (Fig. 2A–C). Among the somatic cell clusters, three subclusters of Sertoli cells, and two subclusters of Leydig cells were obtained (Supplementary Fig. S1A, B).

#### Identification of subclusters and the precise effects of Sox30 on spermatogonia

A total of 304 Sox30^+/+^ and 513 Sox30^−/−^ spermatogonia were reclustered, and the subclusters were annotated. The subcluster of spermatogonia named spermatogonia_1 comprises a mixture of undifferentiated primitive type A spermatogonia and differentiated type A_1-4_ spermatogonia, as they express several spermatogonial stem cell markers, such as *Uchl1*, *Zbtb16* (aka *Plzf*), *Foxf1* and *Scd4*, and early differentiating markers of type A_1-4_ spermatogonia, such as *Ccnd2*, *Dmrt1* and *Sohlh1* [13, 42] (Fig. 2A; Supplementary Tables S2, S3). On the basis of the expression of these markers, spermatogonia_1 was identified as type A spermatogonia. In addition, the cadherin 15 (Chd15) gene may be a new marker for type A spermatogonia. The subcluster of spermatogonia, named spermatogonia_2, included *Stra8-* and *Dmrtb1-*positive cells and expressed high levels of *Stxbp51*, *Sdk1*, and meiotic genes such as *Rad51ap2*, *Sycp3*, *Prdm9* and *Rsph1*, suggesting that spermatogonia_2 should be type B/preleptotene (type B/prelep) spermatogonia (Fig. 2A; Supplementary Tables S2, S3). The results of the quantitative analyses of type A and type B/prelep spermatogonia revealed that the proportion of type A spermatogonia was increased, whereas the proportion of type B/prelep spermatogonia was decreased in the Sox30-null mice (Fig. 2A), suggesting that Sox30-null likely affects the differentiation of spermatogonia.

#### Identification the subclusters and the precise role of Sox30 in spermatocytes

A total of 1414 Sox30^+/+^ and 3079 Sox30^−/−^ spermatocytes were reclustered, and subclusters of spermatocytes were identified. Two spermatocyte subclusters, named spermatocyte_1 and _3, were produced after subclustered in Sox30^+/+^ testes. In contrast, four spermatocyte subclusters, named spermatocyte_1, _2, _4 and _5, were observed after subclustering in Sox30^−/−^ testes, and Sox30^−/−^ testes clearly gave rise to three independent spermatocyte subclusters (i.e., spermatocyte_2, _4 and _5) that superseded spermatocyte_3 from Sox30^+/+^ testes (Fig. 2B). Earlier genetic experiments have identified many markers for distinguishing leptotene (e.g., *Dmc1*, *Prss50*, *Prdm9*, *Terb1*, *Arl6ip1* and *Nol8*), zygotene (e.g., *Rad51ap2*, *Hfm1*, *Stra8*, *Spata22* and *Meiob*), leptotene-to-zygotene (*Sycp1*, *Sycp3*, *Tfdp1*, *Adad2, Prss50* and *Tex101*), pachytene (e.g., *Cdc42ep3*, *Tdrkh*, *Piwil1*, *Rsph1*, *Hsp90b, Hormad1* and *Calm2*) and diplotene-to-division (e.g., *1700001F09Rik*, *BC061237*, *Fzr1*, *Ssxb1*, *Ccnb1* and *Gm3453*) spermatocytes [9, 13, 42–44]. A group of prototypical meiotic gene signatures, such as *Arl6ip1*, *Nol8*, *Cdc42ep3*, *Piwil1*, *Hsp90b* and *Calm2*, comprising leptotene to pachytene were found in spermatocyte_1, along with the expression of a panel of mitochondrial cytochrome C oxidase (Mt-COX) and NADH dehydrogenase genes (*mt-Co2*, *mt-Co1*, *mt-Co3*, *mt-Nd1*), suggesting that spermatocyte_1 should be meiosis I (leptotene-to-pachytene) spermatocytes (Fig. 2B; Supplementary Tables S2, S3), i.e., primary spermatocyte (pSPC). Spermatocyte_3 consisted of *Calm2*-, *Rec8*-, and *E2f5*-positive cells and highly expressed many genes that are responsible for postmeiotic haploid development, such as *Dsg1a*, *Spaca1*, *Pex5l*, *Prm1*, *Prm2*, *Tnp1*, *Tnp2*, and *Smcp* (Fig. 2B; Supplementary Tables S2, S3), suggesting that spermatocyte_3 is likely the second spermatocytes (sSPC). In addition, *Gm34678* and *4933402* *C06Rik* seemed to be new markers for sSPC. Spermatocyte_2 cells were *Rad51ap2-*, *Piwil1-* and *Cdc42ep3-*negative, but specifically expressed *Galnt6*, *G630093K05Rik* and *Gm10775*, and a series of early-round-spermatid developmental genes (such as *Prm1*, *Prm2*, *Tnp1*, *Tnp2*, *Smcp* and *Rnf151*) were significantly downregulated (Fig. 2B; Supplementary Tables S2, S3). However, synaptonemal complex protein 3 (*Syce3*) and a group of genes involved in spindle or cilium assembly (*Kif11*, *Pibf1*, and *Mzt1*), and cell division (such as *Brsk2, Ccnd3*, *Ccnt1*, *Cdc26* and *Sde2*) were upregulated in spermatocyte_2 (Fig. 2B; Supplementary Tables S2, S3). As these patterns are consistent with those of spermatocytes corresponding to diplonema-division cells at the meiosis I metaphase stage [45–47], spermatocyte_2 should be meiosis I metaphase spermatocytes (mMSPCs). Spermatocyte_4 consisted of *Tdrkh-*, *Syce3-* and *Rad51ap2-*positive cells and highly expressed *Vgf* and *Gm26870*, along with low levels of (early) pachytene marker genes (*Cdc42ep3*, *Hspa5*, and *Piwil1*) as well as specifically low levels of marker genes for early round spermatid development, such as *Prm1* and *Acyp1* (Fig. 2B; Supplementary Tables S2, S3), suggesting that spermatocyte_4 are likely zygotene-like spermatocytes (zSPCs). Spermatocyte_5 expressed *Ptafr*, *Spink1* and *Ccr3*, but lacked pachytene marker genes, such as *Rsph1*, *Cdc42ep3*, *Piwil1*, *Hspa5*, *Hormad1* and *Prss42*, along with high levels of diplotene genes such as *Ace*, *1700001F09Rik* and *BC061237* and acrosome formation genes, e.g., *Acrv1*, *Spag6*, *Tekt1*, *Prr27*, and *1700016D06Rik* (Fig. 2B; Supplementary Tables S2, S3), suggesting that spermatocyte_5 are probably diplotene-like spermatocytes (dSPCs) with abnormally developed acrosomes.

In contrast with those in Sox30^+/+^ testes, the proportion of pSPCs (spermatocyte_1) was increased, no normally developing sSPCs (spermatocyte_3, the proportion of which was 0.78571 in Sox30^+/+^ mice) were observed, and three new spermatocyte sub-clusters: mMSPCs (spermatocyte_2), zSPCs (spermatocyte_4) and dSPCs (spermatocyte_5), were found in Sox30^−/−^ mice. The mMSPCs (spermatocyte_2) in Sox30^−/−^ mice seemed to be closer to the sSPCs (spermatocyte_3) in Sox30^+/+^ mice, but lacked the early developmental signal for postmeiotic haploids while highly expressing sperm acrosome genes. The proportion of mMSPCs (spermatocyte_2) was only 0.38423, and the remaining spermatocytes were likely arrested at the meiotic stage in Sox30^−/−^ mice. The proportion of pSPCs (spermatocyte_1) shared with Sox30^+/+^ individuals was increased by approximately 76% in Sox30^−/−^ mice (0.21429 in Sox30^+/+^ mice vs. 0.37789 in Sox30^−/−^ mice), and the proportions of zSPCs (spermatocyte_4) and dSPCs (spermatocyte_5) were 0.13462 and 0.10326 in Sox30^−/−^ mice, respectively (Fig. 2B). These results suggest that Sox30-null clearly causes spermatocyte arrest at the early meiotic phase.

#### Identification of subclusters and the precise effect of Sox30 in spermatids

A total of 7522 Sox30^+/+^ and 756 Sox30^−/−^ spermatids were obtained and reclustered, and the subspermatid clusters were annotated. The latent Mt-COX-related genes *mt-Co3* and *mt-Co2* may presumably reflect requirements for the earliest round of spermatid alterations in energy demand, as these cells divided from the final stages of meiosis [48, 49], and these genes were clearly detected in the spermatid_1 cluster (Fig. 2C; Supplementary Tables S2, S3), suggesting that spermatid_1 is likely round spermatids (rSTs). The maximal expression of the postmeiotic activation of the transition protein-encoding gene *Tnp2*, and the protamine genes *Prm1* and *Prm2*, which are highly expressed in the spermatid_2 clusters (Fig. 2C; Supplementary Tables S2, S3), is required for sperm DNA packaging in elongating spermatid (eST) development from round spermatids [13, 43], indicating that spermatid_2 should be the eSTs. On the basis of the sparse quantity of spermatid_3 and spermatid_4 (the proportion of combined spermatids was ≤ 7% of the total spermatids) and the possibility of teratospermia [50], the spermatid_3 and spermatid_4 clusters could be defined as abnormal spermatids when combined with the molecular features. Spermatid_3 expressed *Ahnak*, *Tshz2* and lncRNA *Malat1* and the seminal plasma-specific marker gene *Ecm1* (extracellular matrix protein 1), which is responsible for sperm nutrition and transport [51], as well as a group of mitochondrial genes (such as *mt-Co1*, *mt-Co2*, *mt-Nd4*, *mt-Cytb* and *mt-Atp6*) and apoptotic genes (such as *Fos*, *Fosb*, *Gsn*, *Jun* and *Junb*), while lacking *Tnp2*, *Prm1* and *Prm2* (Fig. 2C; Supplementary Table S2). Spermatid_4 expressed several genes (such as *Insl3*, *Ptgds*, *Lcn2*, *Agt* and *Clu*) that are frequent markers of Leydig cells or Sertoli cells, and these genes (encoding proteins) are supposed to regulate sperm maturation and transport in the epididymis (Fig. 2C; Supplementary Table S2).

In contrast with that in Sox30^+/+^ testes, the proportion of rSTs (spermatid_1) in Sox30^−/−^ testes increased nearly to 1.5 folds (0.54615 in Sox30^+/+^ mice vs. 0.77681 in Sox30^−/−^ mice), whereas the proportion of eSTs (spermatid_2) in Sox30^−/−^ testes decreased almost 0.43 folds (0.29024 in Sox30^+/+^ mice vs. 0.16667 in Sox30^−/−^ mice, Fig. 2C). The proportion of abnormal spermatids (spermatid_3: 0.05215 in Sox30^+/+^ vs. 0.03913 in Sox30^−/−^ mice; spermatid_4: 0.01145 in Sox30^+/+^ vs. 0.01739 in Sox30^−/−^ mice) seemed to exhibit no obvious changes between Sox30^+/+^ and Sox30^−/−^ mice (Fig. 2C). These data along with the enormous variation in total sperm count between Sox30^+/+^ mice and Sox30^−/−^ mice, indicate that Sox30-null strongly affects spermatid formation.

#### Identification the sub-clusters and Sox30 exact role in Sertoli cells

A total of 417 Sox30^+/+^ and 533 Sox30^−/−^ Sertoli cells were reclustered, and the subclusters were identified. In combination with known mature Sertoli cell markers (e.g., *Clu* (aka *Sgp-2*), *Gata1*, *Wt1*, *Cstl*, *Cst9*, and *Cst12*) and immature Sertoli cell markers (e.g., *Nr5a1*, *Krt18, Amh* and *Thra*) [52–57], high expression of *Cts9*, *Cts12*, *Clu*, *Wt1* and *Cstl* was observed in Sertoli cells_1 (Supplementary Fig. S1A; Tables S2, S3), suggesting that Sertoli cells_1 should be defined mature Sertoli cells (mSCs, i.e., these cells no longer undergo mitosis). In addition, *Glul*, *Nudt19* and *Wfdc6a* may be new markers for mSCs. Sertoli cell_2 expressed mitochondrion organization genes such as *C1qbp* and *Ndufa3* and the cell adhesion gene *Cldn34b3* at high levels, but expressed *Clu*, *Cstl*, and *Cst12* at low levels (Supplementary Fig. S1A; Supplementary Table S2). Moreover, high levels of cell cycle/division regulation genes *Syce1*, *Syce3*, *Ccdc83*, *Ccnb1*, *Ppm1d* and *Kif2a* were observed (Supplementary Fig. S1A; Supplementary Tables S2, S3), indicating Sertoli cells_2 could be considered as immature-like Sertoli cells (imSCs). In Sertoli cells_3, most markers of mature Sertoli cells were highly expressed, also and multiple genes related to apoptosis and inflammation such as *Fos*, *Fosb*, *Jun*, *Cxcl1* and *Nfkbia*,were highly expressed (Supplementary Fig. S1A; Supplementary Table S2), implying that Sertoli cells_3 may be apoptotic/senescent Sertoli cells (seSCs). In addition, this population highly expressed *Cdhr5*, *Nfkbiz* and *Nt5e*, which may be potential markers for seSCs. Compared with that in Sox30^+/+^ testes, the proportion of imSCs (Sertoli cells_2) was slightly greater (0.42206 in Sox30^+/+^ mice vs. 0.54784 in Sox30^−/−^ mice), whereas the proportion of mSCs (Sertoli cells_1) was slightly lower (0.54916 in Sox30^+/+^ mice vs. 0.44465 in Sox30^−/−^ mice), and the proportion of seSCs (Sertoli cells_3) was largely decreased (0.02878 in Sox30^+/+^ mice vs. 0.00750 in Sox30^−/−^ mice) in Sox30^−/−^ mice (Supplementary Fig. S1A). These results reveal that Sox30 may regulate the maturation of testicular Sertoli cells.

#### Identification of subclusters and accurate Sox30 effect in Leydig cells

The Leydig cells were classified into two subclusters, Leydig cell_1 and Leydig cell_2. The expression of the functional marker genes *Insl3*, *Fabp3*, *Rbm47* and *Serpina5*, and the maturation-promoting marker genes *Hsd3b6*, *Hsd17b3*, *Vcam1* and *Star*, was significantly upregulated in Leydig cells_2 (Supplementary Fig. S1B; Supplementary Tables S2, S3). In particular, high expression of *Cyp26b1* and *Aldh1a1*, and reduced expression of *Sf1* (aka *Nr5a1*), were observed in Leydig cells_2 (Supplementary Fig. S1B; Supplementary Table S2). In contrast, the immature marker genes insulin-like growth factors (IGF) *Igf1*, *Igfbp6* and *Igfbp*3 were highly expressed in Leydig cells_1, which were marked by *Tnxb*, *1700125H20Rik* and *Tmem210* (Supplementary Fig. S1B; Supplementary Table S2). Among these common Leydig cell markers [58–62], Leydig cells_2 possessed the functional features of mature Leydig cells (mLCs), and Leydig cells_1 possessed the characteristics of immature Leydig cells (imLCs). The results of the quantitative analyses of the two subclusters of Leydig cells revealed that the proportion of imLCs (Leydig cells_1) was decreased (0.98077 in Sox30^+/+^ mice vs. 0.79592 in Sox30^−/−^ mice), and the proportion of mLCs (Leydig cells_2) was clearly increased (0.01923 in Sox30^+/+^ mice vs. 0.20408 in Sox30^−/−^ mice) in Sox30^−/−^ mice (Supplementary Fig. S1B). These data demonstrate that Sox30 clearly affects the mature phenotype of testicular Leydig cells.

Taken together, our scRNA-seq data reveal that Sox30 significantly impacts the composition and proportion of testicular cell subclusters. In particular, Sox30 deletion clearly altered the composition of spermatocyte subclusters, with an increased proportion of meiotic spermatocytes seemingly without normal postmeiotic cells, which is consistent with the dramatic reduction in sperm cell quantity in Sox30^−/−^ mice. Moreover, Sox30 can regulate the mature phenotype of Sertoli cells and Leydig cells. In addition, Sox30 seems to slightly increase the proportion of type A spermatogonia, which are early differentiated spermatogonia.

### Verification of the major altered testicular cells in Sox30-null mice

**Fig. 3:**
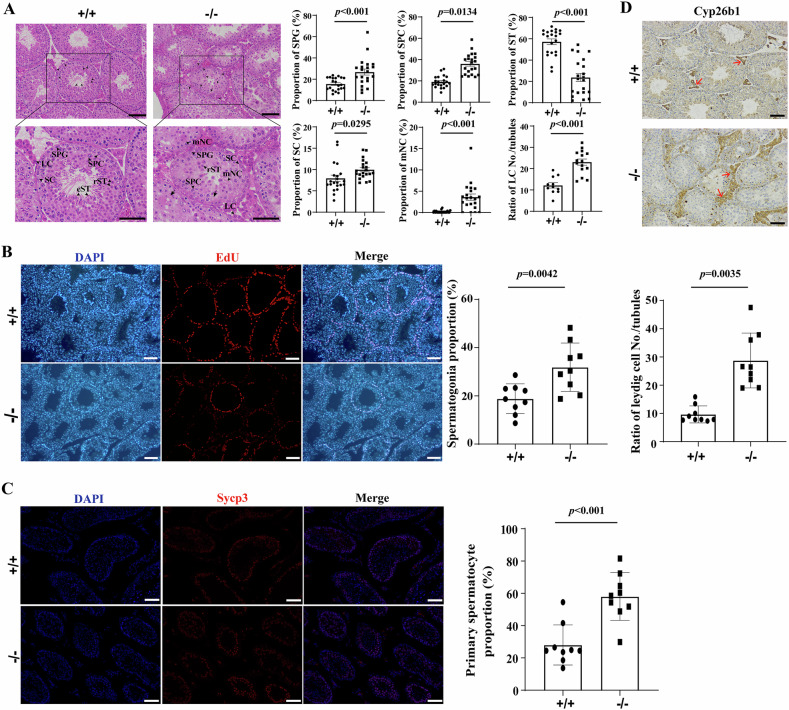
Altered composition proportions of major testicular cells in Sox30-null mice. **A** Histopathological and quantitative analyses of testicular cells in Sox30^+/+^ and Sox30^−/−^ mice at 10 weeks. Left panel: histopathology of testes. Arrows labelled “SPG” indicates spermatogonia, “SPC” indicates spermatocytes, “rST” indicates round spermatids, “eST” indicates elongated spermatid, “SC” indicates Sertoli cells, “LC” indicates Leydig cells, “mNC” indicates multi-nucleated cells, and arrows with tails indicate vacuoles and suspected apoptotic multi-nucleated cells or spermatogenic cells. Right panel: quantitative analyses of major testicular cells. Cell proportions were quantified based on an average of 21 complete seminiferous tubules from 9 random fields (*n* = 21), and normalized by the total cell number per seminiferous tubule. Leydig cell numbers were quantified based on an average of 15 random fields and normalized by the number of seminiferous tubules in the area. **B** Cell proliferation of spermatogonia measured by 5-ethynyl-2’-deoxyuridine (EdU) assays in testes of mice at 10 weeks. Cell proliferation of spermatogonia was quantified based on an average 9 seminiferous tubule from 3 random fields and normalized by the total cell number per seminiferous tubule. **C** Primary spermatocytes marked by Sycp3 in adult testes. The proportion of primary spermatocyte quantified based on an average 9 seminiferous tubule from 3 random fields and was normalized by the total cell number per seminiferous tubule. **D** Leydig cells marked by Cyp26b1 in adult testes. Brown precipitate indicated by red arrows represent immunopositive cells. The number of Leydig cells per seminiferous tubule was quantified based on an average of 9 random fields. Scale bars are 50 µm.

To further confirm the key results of scRNA-seq, pathological H&E staining of testes of different genotypes and quantitative analyses were performed. In line with the results of scRNA-seq, increased proportion of spermatogonia (SPGs), spermatocytes (SPCs), Sertoli cells (SCs) and Leydig cells (LCs), reduced proportion of spermatids (STs), and the absence of elongating spermatids were observed in Sox30^−/−^ mice (Fig. 3A, Supplementary Table S4). Moreover, many multi-nucleated cells (mNCs), vacuoles and suspected apoptotic multinucleated cells or spermatogenic cells were found in the seminiferous tubules of Sox30^−/−^ mice (Fig. 3A, Supplementary Table S4). To further verify the change in the proportion of spermatogonia, assay to detect the proliferation of spermatogonia was performed using 5-ethynyl-2’-deoxyuridine (EdU). The number of complete seminiferous tubule cells was calculated from three random views of each mouse and from three independent mice of each genotype (*n* = 9). A significant increase in the proportion of spermatogonia (EdU-positive cells) was observed in the seminiferous tubules of Sox30^−/−^ mice (*p* < 0.01, Fig. 3B, Supplementary Table S4). Immunostaining for Sycp3 (a marker of primary spermatocytes) was performed on testicular tissues to evaluate the number of spermatocytes in Sox30^+/+^ and Sox30^−/−^ mice, and the proportion of primary spermatocytes in seminiferous tubules clearly increased nearly 2-fold in Sox30^−/−^ mice (*p* < 0.001, Fig. 3C, Supplementary Table S4). Leydig cells were also significantly increased according to the results of the quantitative immunostaining analysis of Cyp26b1 (a marker of Leydig cell) in Sox30^−/−^ mice (*p* < 0.01, Fig. 3D, Supplementary Table S4). These histological results strongly confirm the alterations in major germ cells and somatic cells from the scRNA-seq data of Sox30^−/−^ mice.

#### Roles of Sox30 in the differentiation of testicular cells

**Fig. 4:**
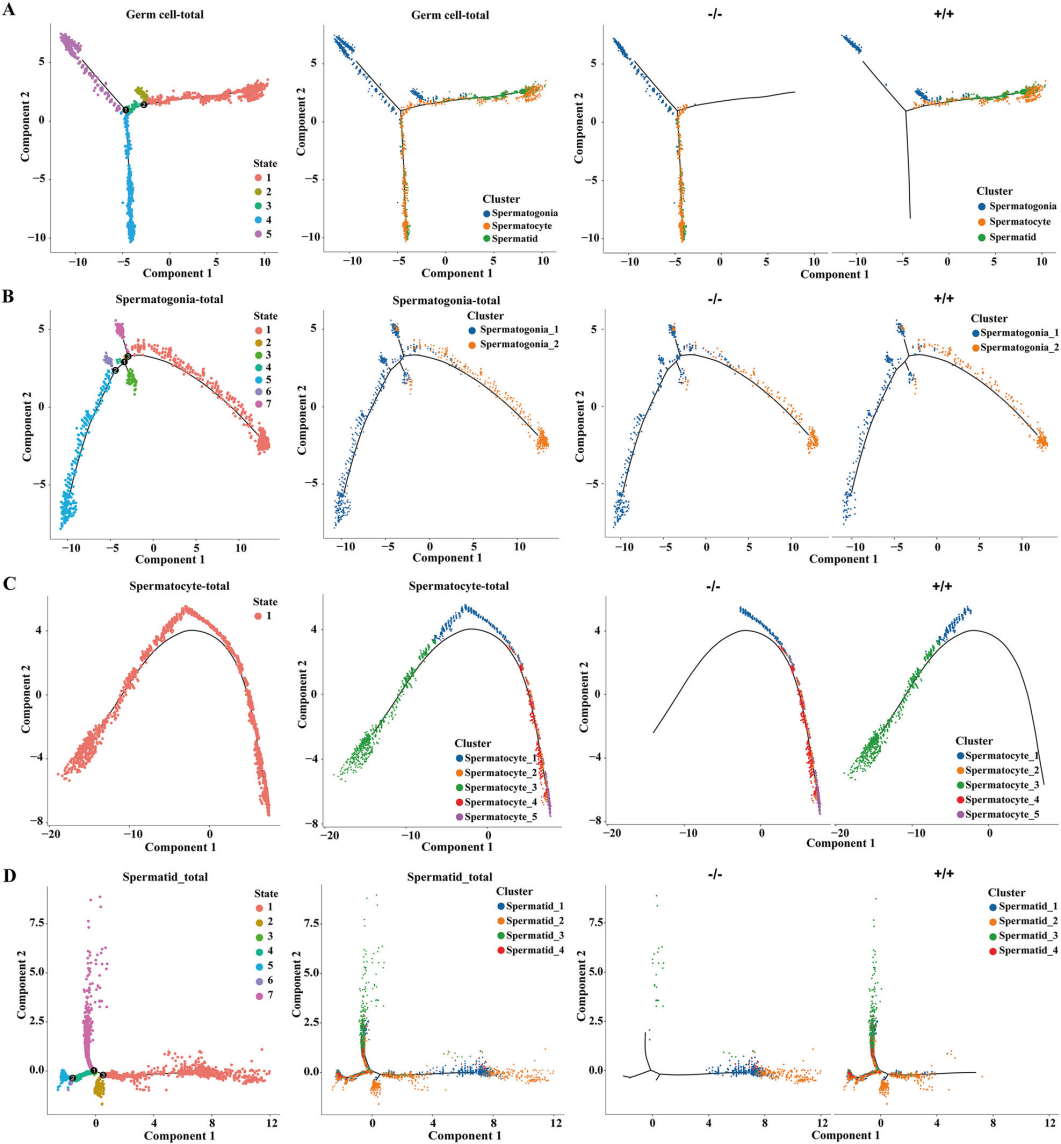
Development trajectories of major testicular cells in Sox30^+/+^ and Sox30^−/−^ mice. **A** From left to right: cell trajectories of integrated germ cells ordered by cell state (left) and integrated germ cells (middle), Sox30^−/−^ (−/−) germ cells, Sox30^+/+^ (+/+) germ cells coloured according to UAMP cell clusters (right). From left to right: cell trajectories with integrated spermatogonia (**B**) / spermatocytes (**C**) / spermatids (**D**) ordered by cell state (left), integrated spermatogonia (**B**) / spermatocytes (**C**) / spermatids (**D**) sub-cell clusters (middle), Sox30^−/−^ (−/−) spermatogonia (**B**) / spermatocytes (**C**) / spermatids (**D**), Sox30^+/+^ (+/+) spermatogonia (**B**) / spermatocytes (**C**) / spermatids (**D**) sub-clusters coloured according to UAMP cell clusters (right).

To predict the roles of Sox30 in major germ cell and somatic cell fates, pseudotime trajectory reconstruction was performed to model the developmental relationships among spermatogonia, spermatocytes and spermatids, Sertoli cells and Leydig cells. The reconstructed pseudotime trajectory clearly corroborated the transition of spermatogonia to spermatocytes and spermatids (Fig. 4A). Interestingly, the pseudotime trajectory began with spermatogonia and then split into two main spermatocyte branches with Sox30^−/−^ spermatocytes and Sox30^+/+^ spermatocytes placed at divergent ends as two differentiated cell types (Fig. 4A). Few spermatids were found at the terminal end of the trajectory in Sox30^−/−^ testicular cells (Fig. 4A), which further corroborated the decrease in the number of spermatids in Sox30-null mice. No marked variation was observed in the developmental trajectories of the two spermatogonia subclusters in Sox30-null mice (Fig. 4B). However, an opposite developmental trajectory was shown for the spermatocyte subclusters in Sox30^−/−^ mice compared with Sox30^+/+^ individuals (Fig. 4C), followed by incomplete development of spermatid subclusters, especially the altered distribution of trajectories in rSTs (spermatid_1) and eSTs (spermatid_2) in Sox30^−/−^ mice (Fig. 4D). No significant differences in the developmental trajectories of subclusters of Sertoli cells and Leydig cells were detected between Sox30^−/−^ and Sox30^+/+^ mice (Supplementary Figs. S2A, S2B). The single-cell data reveal that Sox30-null primarily alters the developmental trajectories of spermatocytes in testicular cells, causing substantial loss of spermatid clusters.

#### The involvement of Sox30 in the cell-to-cell communication of testicular cells

**Fig. 5:**
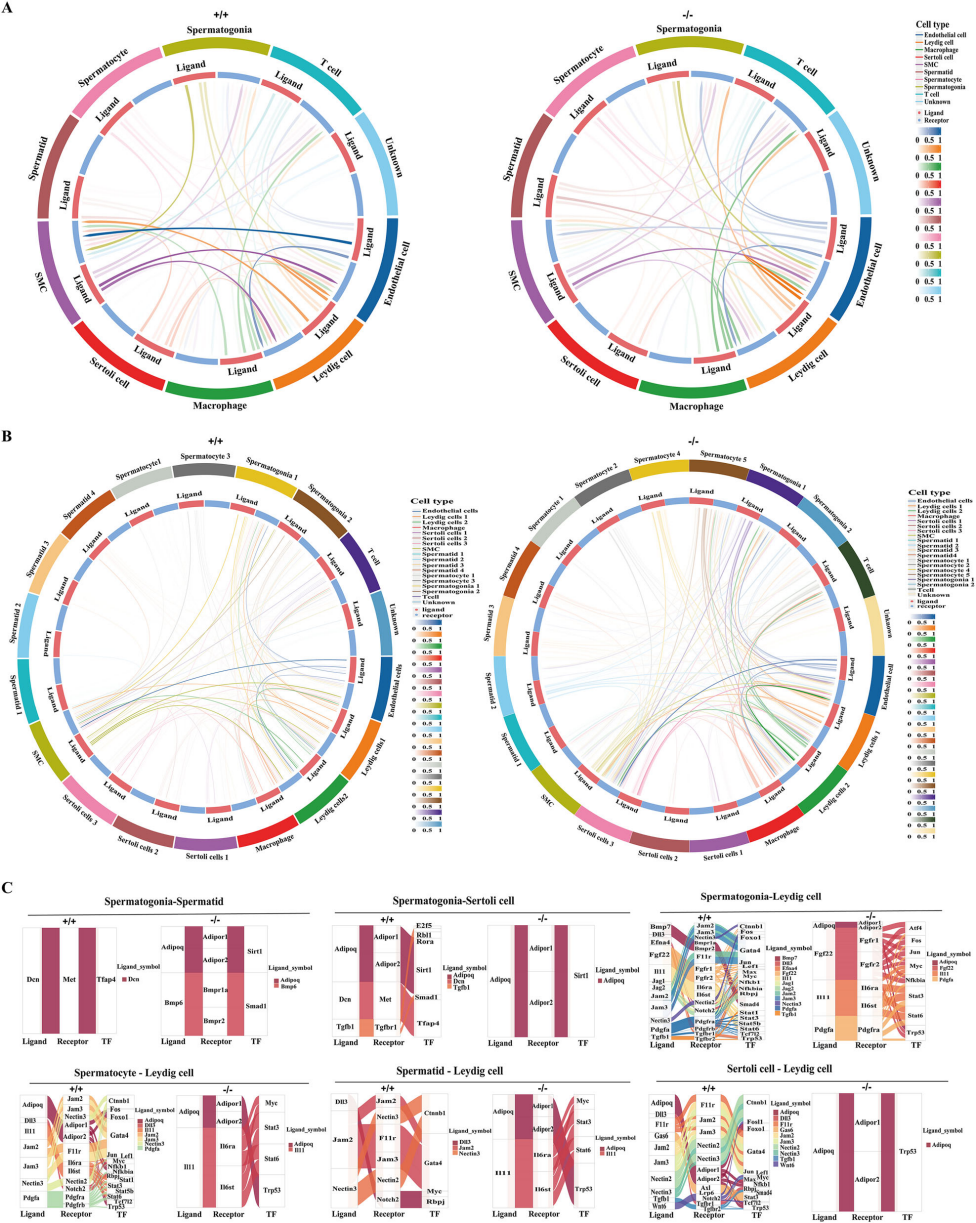
Cell communication characteristics and ligand-receptor interactions between testicular cells influenced by Sox30. **A** Cellchat analysis of ligand-receptor pairs across ten cell clusters. Cellchat analysis shows the predicted number of ligand-receptor and the average interaction strength (colour gradation) in Sox30^+/+^ (+/+) and Sox30^−/−^ (−/−) mice. The red arc in the inner circle represents ligands, and the blue arc represents receptors. **B** Cellchat analysis of ligand-receptor pairs across sub-cell clusters of major testicular cells. CellChat analysis of ligand-receptor pairs across sub-cell clusters in Sox30^+/+^ (+/+) and Sox30^−/−^ (−/−) mice. **C** Sankey plot of ligand-receptor interactions and crucial TF networks. Sankey plot showing ligand-receptor interactions and the network of crucial TFs among the following cell pairs: spermatogonia-spermatid, spermatogonia-Sertoli cell, spermatogonia-Leydig cell, spermatocyte-Leydig cell, spermatid-Leydig cell and Sertoli cell-Leydig cell in Sox30^+/+^ (+/+) and Sox30^−/−^ (−/−) mice, respectively. Each ligand is assigned a different colour, and the line width represents the strength of the interaction.

The communication between germ cells and various surrounding cells is the key event in testicular architecture and sperm production [5, 63]. To investigate the roles of Sox30 in maintaining the spermatogenic microenvironment, we analysed the communication between germ cell and germ cell, between germ cell and somatic cell, and between somatic cell and somatic cell for major testicular cells via CellChat. Sox30 was clearly involved in the strength as well as the amount of ligand‒receptor signalling between testicular cells. The outgoing and incoming interactions of all the major germ cells and somatic cells seemed to be altered in Sox30-null mice (Fig. 5A). Sox30-null attenuated the communication between spermatogonia and Sertoli cell, between spermatogonia and Leydig cell, between spermatogonia and SMC and between spermatogonia and macrophage, but slightly strengthened the communication between spermatogonia and spermatids and between spermatogonia and endothelial cells (Fig. 5A). Spermatocytes in Sox30-null mice presented increased interactions with endothelial cells but decreased interactions with Leydig cells and SMCs (Fig. 5A). Spermatids in Sox30-null mice displayed slightly enhanced communication with Leydig cells, and increased communication with endothelial cells (Fig. 5A). Sertoli cells in Sox30-null mice displayed attenuated communication to Leydig cells, SMCs and endothelial cells, and Leydig cells in Sox30-null mice displayed attenuated communication to SMCs but increased communication with T cells and endothelial cells (Fig. 5A).

A significantly altered interaction among subclusters of major germ cells and somatic cells was observed in Sox30^−/−^ mice. Compared with Sox30^+/+^ mice, spermatogonia_1 in Sox30-null mice displayed less communication with Leydig cell_1 and Sertoli cell_2 but intensive communication with Spermatid_2, Spermatid_4, Leydig cell_2, Sertoli cell_3 and endothelial cells. Spermatogonia_2 in Sox30-null mice presented enhanced communication with Leydig cell_1 and Sertoli cell_3 (Fig. 5B). Spermatocyte_1 in Sox30-null mice displayed enhanced communication with Leydig cell_2 and Sertoli cell_3; moreover, Spermatocyte_2, _4 and _5 displayed intensive cross-talk with Sertoli cell_3, Leydig cell_1 and Leydig cell_2 (Fig. 5B). Spermatid_1 in Sox30-null mice presented slightly increased communication with Sertoli cell_3; Spermatid_2 presented increased communication with Sertoli cell_3, Leydig cell_1 and Leydig cell_2 in Sox30-null mice; and Spermatid_3 in Sox30-null mice presented slightly increased communication with Sertoli cell_3, Leydig cell_2, endothelial cell and T cells. Spermatid_4 in Sox30-null mice also presented increased communication with Sertoli cell_3, Leydig cell_2and endothelial cells (Fig. 5B). Sertoli cell_3 displayed enhanced communication with Sertoli cell_1, Leydig cell_2 and endothelial cells in Sox30-null mice (Fig. 5B). Leydig cell_1 in Sox30-null mice displayed enhanced communication with T cells, and Leydig cell_2 in Sox30-null mice showed enhanced communication with endothelial cells (Fig. 5B). In addition, the communication of SMC-endothelial cells, SMC-Spermatogonia_1/Spermatogonia_2, SMC-Leydig cell_1/Leydig cell_2, and SMC-Sertoli cell_1/Sertoli cell_2/Sertoli cell_3 was also attenuated in Sox30-null mice (Fig. 5B).

Further analyses of each cell‒cell communication revealed that the number of receptor and ligand bindings, and the number of transcription factors (TFs) were increased in the spermatogonia-spermatid, but were significantly decreased in the spermatogonia-Sertoli cell, spermatogonia-Leydig cell, spermatocyte-Leydig cell, spermatid-Leydig cell and Sertoli cell-Leydig cell in Sox30^−/−^ mice (Fig. 5C). In addition, the number of receptor/ligand bindings and TFs were also significantly decreased in the spermatogonia-SMC, spermatocyte-SMC, endothelial cell-SMC and SMC-Leydig cell (Supplementary Fig. S3). These results indicate that Sox30-null can clearly alter the intercellular cross-talk of the testicular cells, especially the cross-talk among germ cells, Sertoli cells, Leydig cells and SMC.

### Effects of Sox30 on transcription factor regulatory networks in the testis

**Fig. 6:**
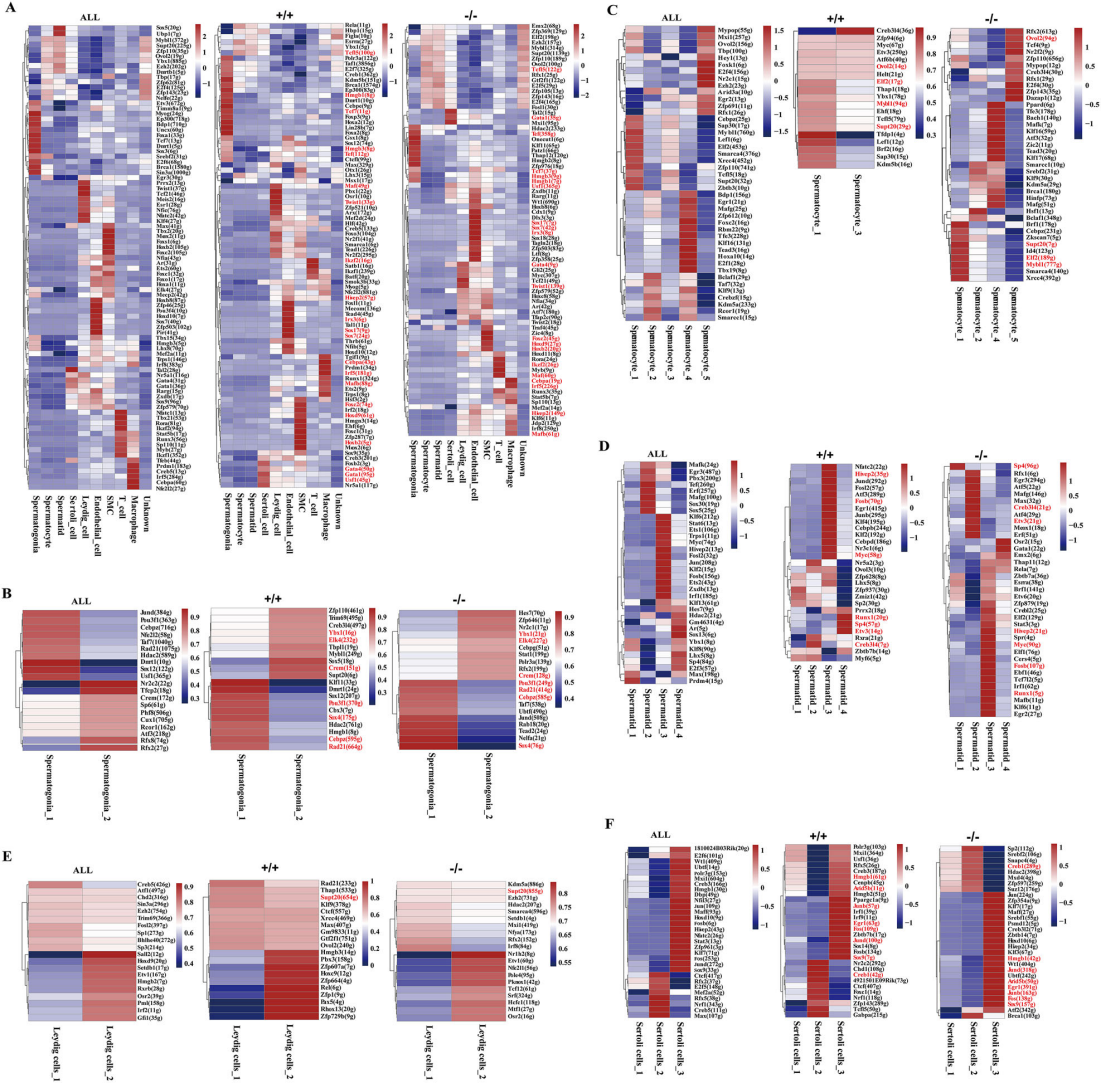
Detailed characterizations of transcription factor regulatory networks in testicular cells of Sox30^+/+^ and Sox30^−/−^ mice. **A** From left to right panel: heatmaps showing the top 10 regulon activities of TFs in the ten clusters of integrated testicular cells (left), Sox30^+/+^ (+/+) testicular cells and Sox30^−/−^ (−/−) testicular cells. From left to right panel: heatmaps showing the top 10 regulon activities of TFs in spermatogonia sub-clusters (**B**), spermatocyte sub-clusters (**C**), spermatid sub-clusters (**D**), Leydig cell subclusters (**E**) and Sertoli cell (**F**) of integrated spermatogonia/spermatocyte/spermatid/Leydig cell/Sertoli cell subclusters (left), these sub-clusters in Sox30^+/+^ (+/+) mice, and these subclusters in Sox30^−/−^ (−/−) mice, respectively. All co-expressed TFs in both Sox30^+/+^ mice and Sox30^−/−^ mice are labelled in red.

To investigate changes in transcription factors (TFs), we used SCENIC to evaluate TF regulon activity in each testicular cell type and construct TF-centred gene coexpression networks. Compared with Sox30^+/+^ mice, the types and activities of TFs in different cells markedly changed after Sox30 loss, and the activities of only a few key TFs labelled in red seemed to be unaffected by Sox30. In spermatogonia, the regulon activities of *Taf1*, *E2f7*, *Creb1*, *Kdm5b, Brca1*, *Lin28b Ep300*, *Foxp3*, *Cebpe*, *Dmrt1*, *Foxa2, etc*., were downregulated, whereas the regulon activities of *Onecut1*, *Klf11*, *Patz1*, *Thap12*, *Hmgb2*, *Hdac2* and Zfp976 were upregulated in Sox30^−/−^ mice (Fig. 6A). Most of those altered TFs play crucial roles in the cell cycle (*Taf1*, *E2f7*, *Creb1*, *Lin28b*, and *Klf11*) and in germ cell development and differentiation (*Dmrt1*, *Foxa2*, *Sox12*, *Cebpe*, and *Hmgb2*), which may correspond to an imbalance in the proliferation and differentiation of spermatogonia. In spermatocytes, the regulon activities of *Rela*, *Hbp1*, *Esrra*, *Ybx1* and *Lhx3* were downregulated in Sox30^−/−^ mice, whereas the abundant regulon activities of *Emx2*, *Zfp369*, *Elf2*, *Ezh2*, *Mybl1*, *Zfp110*, *Ovol2*, *E2f5, E2f4*, *Gtf2f1*, *Fosl1*, *Zfp143, etc*., whose functions are primarily involved in cell proliferation and the meiotic cycle, were dysregulated in Sox30^−/−^ mice (Fig. 6A). These altered TF features were closely associated with abnormal spermatocyte division. In spermatids, the regulon activities of altered TFs appeared to substantially overlap with those of spermatocytes in Sox30^−/−^ mice. Moreover, the regulon activities of *Figla* and *E2f7* were increased, whereas the regulon activities of *Gtf2f1, E2f5*, *E2f4* and *Fosl1* seemed to be further activated in Sox30^−/−^ mice (Fig. 6A; Supplementary Table S5). These persistently activated TFs suggest the abnormal development status of spermatids in Sox30^−/−^ mice. Interestingly, the regulatory activities of altered TFs in multi-nucleated cells (unknown cell cluster) seemed to largely overlap with those of spermatocytes and spermatids in Sox30^−/−^ mice (Fig. 6A; Supplementary Table S5), which is consistent with the property of this cell cluster possessing the characteristics of both spermatocytes and spermatids. In Sertoli cells, the regulon activities of *Gata1*, *Gata4* and *Usf1* showed little change, whereas the regulon activities of *Nr5a1* and *Foxb2*, which may play roles in cell maturation and differentiation, were dysregulated in Sox30^−/−^ mice (Fig. 6A; Supplementary Table S5). In addition, the regulon activities of *Tal2* and *Mxi1* were upregulated, whereas the regulon activities of *Mef2a*, *Atf7*, *Traf4*, *Tcf7* and *Tef* were downregulated in Sertoli cells from Sox30^−/−^ mice. In Leydig cells, the regulon activity of *Gata4* appeared to increase, and the regulon activity of *Maf* was almost completely lost after Sox30 loss (Fig. 6A; Supplementary Table S5). More importantly, the regulon activities of the TFs involved in cell differentiation (*Zfp52*, *Arx*, *Mef2d*, *Foxo3*, *Hlf*, *Creb5*, *Nr2f1*, and *Nr2f2*) and proliferation (*Tgif1*, *Tcfl5*, and *E2f7*), and in retinoic acid signalling (*Osr1*, *Sox9*, and *Tead1*) were decreased, whereas the regulon activities of the TFs might involve in cell proliferation (*Gil2*and *Myc*), development process (*Nfia*, *Tcf21*, *Hoxc8*, *Atf7*, and *Twist2*), germline specification and steroid signalling (*Tfap2c*and *Ar*), were highly activated in Sox30^−/−^ mice (Fig. 6A; Supplementary Table S5). These altered TFs seem to be closely associated with changes in the maturation and functional phenotypes of Leydig cells. In addition, the regulatory activities of TFs in other somatic cells, such as SMCs, macrophages, T cells, and endothelial cells, were also strongly affected (Fig. 6A; Supplementary Table S5).

Furthermore, the TF network in each subcluster of major testicular cells was markedly altered in Sox30^−/−^ mice, and those testicular cell subclusters could be distinguished by different groups of TFs. In Spermatogonia_1, the regulon activities of *Pou3f1*, *Sox4*, *Cebpz* and *Rad21* appeared not be influenced, whereas the regulon activities of *Klf11*, *Dmrt1*, *Sox12*, *Cbx3*, *Hdac2* and *Hmgb1*, the majority of which are involved in spermatogenesis and the cell cycle, were dysregulated after Sox30 loss (Fig. 6B). In addition, the regulon activities of *Taf7*, *Ubtf*, *Jund*, *Rab18*, *Tead2* and *Nelfa* were upregulated in Sox30^−/−^ mice (Fig. 6B). In Spermatogonia_2, the regulon activities of *Zfp110*, *Trim69*, *Creb3l4*, *Tbp11*, *Mybl1*, *Sox5* and *Supt20* were dysregulated in Sox30^−/−^ mice (Fig. 6B), and the majority of these TFs play roles in cell apoptosis and differentiation. Moreover, the regulon activities of *Hes7*, *Zfp646*, *Nr2c1*, *Cebpg*, *Stat1*, *Polr3a* and *Rfx2* were upregulated in Sox30^−/−^ mice (Fig. 6B). These dysregulated TFs in Spermatogonia_1/_2 appeared to be clearly associated with the proliferation and differentiation of Spermatogonia_1 (type A spermatogonia) and Spermatogonia_2 (type B spermatogonia). In Spermatocyte_1, the regulon activities of *Zfp94*, *Myc*, *Etv3*, *Atf6b*, *Helt*, *Thap1*, *Ybx1*, *Ehf*, *Tcf15*, *Tfdp1*, *Lef1*, *Sap30* and *Kdm5b*, which are involved mainly in the cell cycle and cell differentiation, were dysregulated, whereas the regulon activities of *Zfp110*, *Brf1*, *Cebpz*, *Zkscn*, *Id4*, *Smarca4* and *Xrcc4*, which are involved primarily in spermatogonia stemness and DNA repair, were upregulated in Sox30^−/−^ mice (Fig. 6C). These disrupted TF regulatory networks are likely involved in the aberrant differentiation of spermatocytes. In Spermatocyte_3 of Sox30^+/+^ mice, the regulon activities of *Creb3l4*, *Zfp94*, *Myc*, *Etv3*, *Atf6b* and *Ybx1*, the bulk of which are involved in cell cycle, differentiation and spermatid development, were activated, whereas the regulon activities of *Tfdp1*, *Lef1*, *Brf2*, *Sap30* and *Kdm5b* were silenced (Fig. 6C). Alterations in the activity of those TFs are likely associated with second meiosis, subsequent cell cycle transitions and early spermatid development. However, Spermatocyte_3 was absent, but was replaced by Spermatocyte_2, Spermatocyte_4 and Spermatoctye_5 in Sox30^−/−^ mice. In Spermatocyte_2, the regulatory activities of *Smarcc1*, *Srebf2*, *Klf9*, *Kdm5a*, *Hsf1* and *Bclaf1*, which function in cell stemness and DNA remodelling and repair, were activated, whereas the regulatory activities of *Mypop* and *Ovol2* were silenced in Sox30^−/−^ mice (Fig. 6C). In Spermatocyte_4, the regulon activities of *Ppard*, *Tfe3*, *Bach1*, *Mafk*, *Klf16*, *Atf3*, *Zic2*, *Tead3*, *Klf17*, *Brca1*, *Hinfp* and *Mafg*, most of which are involved mainly in DNA repair, cell division and stemness maintenance, were activated, whereas the regulon activities of *Rfx2*, *Ovol2*, *Tcf4*, *Nr2f2* and *Zfp110* were silence in Sox30^−/−^ mice (Fig. 6C). In Spermatocyte_5, the regulon activities of *Rfx26*, *Ovol2*, *Tcf4*, *Nr2f2*, *Zfp110*, *Mypop*, *Creb3l4*, *Rfx1*, *E2f4*, *Zfp143*, and *Dazap1* were activated, whereas *Bclaf1*, *Cebpz* and *Kdm5a* were silenced in Sox30^−/−^ mice (Fig. 6C). Most of the dysregulated TFs in Spermatocyte_1-_5 are involved in DNA repair and cell division, which further suggests the abnormal differentiation (meiotic) state of spermatocytes in Sox30^−/−^ mice. In Spermatid_1, the regulon activities of *Sp4*, *Zbtb7a*, *Esrra*, *Brf1*, and *Etv6*, which are involved mainly in cell proliferation and differentiation, were upregulated, whereas the regulon activities of *Zfp628*, *Zfp937*, *Zmiz1*, *Sp2* and *Myf6*, which are involved in spermatogenesis, were dysregulated in Sox30^−/−^ mice (Fig. 6D). In Spermatid_2, the regulon activities of *Rfx1*, *Egr3*, *Atf5*, *Mafg*, *Max*, *Atf4*, *Etv3*, *Meox1* and *Erf*, most of which are involved in cell differentiation, were upregulated, whereas the regulon activities of *Nr5a2*, *Ovol3*, *Rxra* and *Myf6* were dysregulated in Sox30^−/−^ mice (Fig. 6D). In Spermatid_3, the regulon activities of *Emx2*, *Thap11*, *Rela*, *Zbtb*, *Esrra*, *Brf1*, *Etv6*, *Zfp979*, *Crebl2*, *Elf2*, *Stat3*, *Spr*, *Elf1*, *Cers4*, *Ebf1*, *Tcf712*, *Irf1*, *Runx1*, *Mafb*, *Klf6*, and *Egr2*, most of which are involved primarily in early gonadal development, spermatogenesis, the cell cycle and oncogenes, were upregulated, whereas the regulon activities of *Nfatc2*, *Jund*, *Fosl2*, *Atf3*, *Egr1*, *Junb*, *Klf4*, *Cebpb*, *Klf2*, *Cebpd* and *Nr3c1* were dysregulated in Sox30^−/−^ mice (Fig. 6D). In Spermatid_4, the regulon activities of *Osr2*, *Gata1*, *Emx2*, *Thap11* and *Rela* were upregulated, whereas the regulon activities of *Prrx2*, *Runx1*, *Sp4*, *Etv3* and *Zbtb7b* were dysregulated in Sox30^−/−^ mice (Fig. 6D). The altered TFs in the spermatid subclusters strongly indicate the abnormal status of spermatids, especially spermatid_1 (round spermatids) and spermtid_2 (elongated spermatids).

In Leydig cell_1, the regulatory activities of *Kdm5a*, *Ezh2*, *Hdac2*, *Smarca4*, *Setdb1*, *Mxi1*, *Nfya*, *Rfx2*, and *Irf8*, whose functions are involved mainly in chromosome remodelling and cell differentiation, were upregulated, whereas the regulatory activities of *Rad21*, *Thap1*, *Klf9*, *Ctcf* and *Xrcc4*, which function mainly in mitosis and cell differentiation, were dysregulated in Sox30^−/−^ mice (Fig. 6E). In Leydig cell_2, the regulon activities of *Nr1h2*, *Etv1*, *Nfe2l1*, *Pole4* and *Pknox1*, which function mainly in cell proliferation, the cell cycle and functional homoeostasis, were upregulated, whereas the regulon activities of *Zfp729b*, *Rhox13*, *Pax5*, *Zfp1*, *Zfp664*, *Hoxc9*, *Zfp607a*, *Pbx3* and *Hmgb3* were dysregulated in Sox30^−/−^ mice (Fig. 6E). These altered TFs in Leydig cell subclusters are involved in primarily chromosome remodelling, cell growth, the cell cycle and proliferation, which are clearly associated with abnormalities in the Leydig cell population. In Sertoli cell_1, the activities of *Suz12*, *Zfp597*, *Mxd4*, *Hdac2* and *Creb1* were upregulated, whereas the regulon activities of *Polr3g*, *Mxi1*, *Usf1* and *Rfx5*, which are involved mainly in cell function and differentiation, were dysregulated in Sox30^−/−^ mice (Fig. 6F). In Sertoli cell_2, the regulatory activities of *Sp2*, *Srebf2*, *Snapc4*, *Hdac2*, *Mxd4*, *Zfp597*, *Suz12* and *Brca1* were upregulated, whereas the regulatory activities of *Nr2c2*, *Chd1*, *4921501E09Rik*, *Ctcf*, *Foxc1*, *Nrf1 Zfp143*, *Tcf15* and *Gabpa*, which play important roles in cell proliferation and differentiation, were dysregulated in Sox30^−/−^ mice (Fig. 6F). In Sertoli cell_3, the regulon activities of *Jun*, *Zfp354a*, *Klf7*, *Maff*, *Srebf1*, *Psmd12*, *Creb3l2*, *Zbtb14*, *Hoxd10*, *Hivep2*, *Klf3*, *Wt1*, *Ubtf* and *Atf2*, most of which play crucial roles in steroid homoeostasis, cell differentiation and apoptosis, were upregulated, whereas the regulon activities of *Polr3*, *Mxi1*, *Usf1*, *Rfx5*, *Creb3*, *Cenpb*, *Hmgb2*, *Ppargc1a*, *Irf1*, *Irf9*, *Zbtb7b*, *Sox14* and *Fosb*, most of which function mainly in cell immune defence and differentiation, were dysregulated in Sox30^−/−^ mice (Fig. 6F). The altered activities of TFs in Sertoli cell subclusters were clearly associated with Sertoli cell growth, differentiation, and steroid homoeostasis. Taken together, the most pronounced changes in TF types and activities were observed in the spermatocyte and Leydig cell subclusters, followed by the spermatid, spermatogonia and Sertoli cell subclusters. The altered regulon activities of these TFs may partly reveal the specific state of each cell subcluster.

#### Key roles and pathways regulated by Sox30 in major testicular cells

**Fig. 7:**
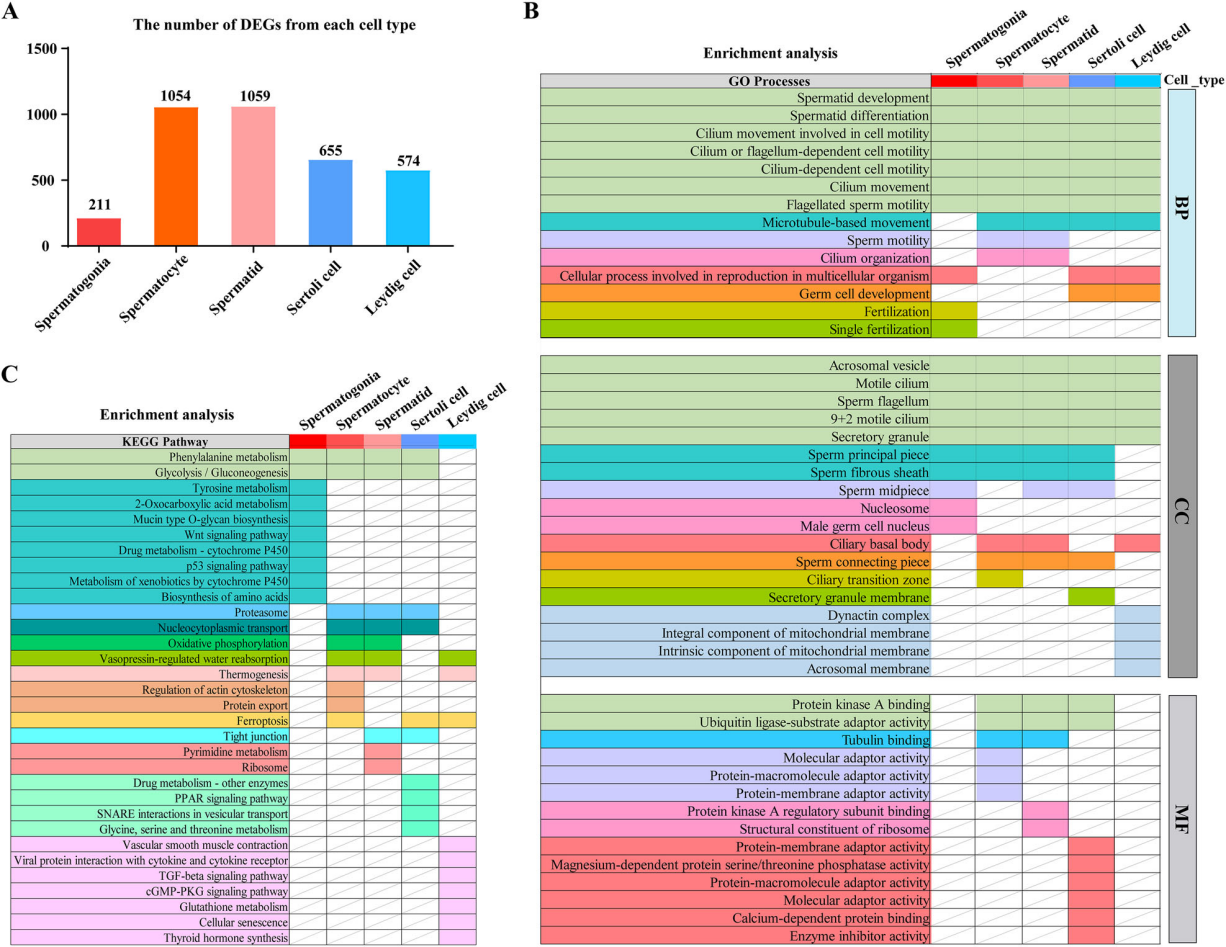
Potential key roles and pathways regulated by Sox30 in major testicular cells. **A** The number of differentially expressed genes (DEGs) identified in major testicular cell clusters from Sox30^−/−^ mice. Functional and mechanistic features of major testicular cell clusters in Sox30^−/−^ mice analysed by GO (**B**) and KEGG (**C**) enrichment analyses. Different GO terms and KEGG pathways with the same colour represent their simultaneous enrichments in various major testicular cell clusters. Uncoloured wells with slash lines indicate unenriched GO terms or KEGG pathways in this a specific cell cluster.

The differentially expressed genes (DEGs) in the major testicular cells of Sox30^−/−^ mice compared with Sox30^+/+^ mice were analysed. A total of 211 DEGs in spermatogonia, 1054 DEGs in spermatocytes, 1059 DEGs in spermatids, 655 DEGs in Sertoli cells, and 574 DEGs in Leydig cells were obtained, and the GO and KEGG enrichment results were analysed for these DEGs (Fig. 7A). The DEGs of each cell type were enriched in similar biological processes, and the DEGs in spermatogonia, spermatocytes, spermatids, Sertoli cells, and Leydig cells were enriched in “spermatid development” (GO:0007286), “spermatid differentiation” (GO:0007286), “cilium movement involved in cell motility” (GO:0060294), “cilium or flagellum-dependent cell motility” (GO:0001539), “cilium-dependent cell motility” (GO:0060285),“cilium movement”(GO:0003341), and “flagellated sperm motility” (GO:0030317) biological processes. “Microtubule-based movement” (GO:0007018) was enriched in these cell types except for spermatogonia, whereas the “fertilization” (GO:0009566) and “single fertilization” (GO:0007338) seemed to be uniquely enriched in spermatogonia. “Sperm motility” (GO:0097722) and “cilium organization” (GO:0097722) were enriched in spermatocytes and spermatids. “Cellular process involved in reproduction in multicellular organism” (GO:0022412) was enriched in spermatogonia, Sertoli cells and Leydig cells, and “germ cell development” (GO:0007281) was enriched in Sertoli cells and Leydig cells (Fig. 7B; Supplementary Table S6, Supplementary Fig. S4).

These DEGs were also enriched in similar cellular components. DEGs from spermatogonia, spermatocytes, spermatids, Sertoli cells and Leydig cells were enriched in “acrosomal vesicle” (GO:0001669), “motile cilium” (GO:0031514), “sperm flagellum” (GO:0036126), “9 + 2 motile cilium” (GO:0097729), “secretory granule” (GO:0030141); “sperm principal piece” (GO:0097228), “sperm fibrous sheath” (GO:0035686) was enriched in spermatogonia, spermatocytes, spermatids and Sertoli cells, and “sperm midpiece” (GO:0097225) was enriched in spermatogonia, spermatids and Sertoli cells; “ciliary basal body” (GO:0036064) was enriched in spermatocytes, spermatids and Leydig cells, and “sperm connecting piece” (GO:0097224) was enriched in spermatocytes, spermatids and Sertoli cells. The “nucleosome” (GO:0000786) and “male germ cell nucleus” (GO:0001673) seemed to be uniquely enriched in spermatogonia, the “ciliary transition zone” (GO:0035869) seemed to be uniquely enriched in spermatocytes, and the “secretory granule membrane” (GO:0030667) was uniquely enriched in Sertoli cells; The “dynactin complex” (GO:0005869), “integral component of mitochondrial membrane” (GO:0032592), “intrinsic component of mitochondrial membrane” (GO:0098573), and “acrosomal membrane” (GO:0002080) appeared to be only enriched in Leydig cells (Fig. 7B; Supplementary Table S6, Supplementary Fig. S4). However, the enrichment of these DEGs in terms of molecular function appeared to be dispersed, with a subset of molecular functions enriched only in spermatocytes, spermatids and Sertoli cells. “Protein kinase A binding” (GO:0051018) and “ubiquitin ligase-substrate adaptor activity” (GO:1990756) were enriched in spermatocytes, spermatids and Sertoli cells, whereas the “tubulin binding” (GO:0015631) was enriched in spermatocytes and spermatids; “molecular adaptor activity” (GO:0060090), “protein-macromolecule adaptor activity” (GO:0030674), and “protein-membrane adaptor activity” (GO:0043495) seemed to be uniquely enriched in spermatocytes, “protein kinase A regulatory subunit binding” (GO:0034237) and “structural constituent of ribosome” (GO:0003735) were enriched in spermatids alone; and “protein-membrane adaptor activity” (GO:0043495), “magnesium-dependent protein serine/threonine phosphatase activity” (GO:0004724), “protein-macromolecule adaptor activity” (GO:0030674), “molecular adaptor activity” (GO:0060090), “calcium-dependent protein binding” (GO:0048306), and “enzyme inhibitor activity” (GO:0004857) were specifically enriched in Sertoli cells (Fig. 7B; Supplementary Table S6, Supplementary Fig. S4).

KEGG enrichment analyses revealed that Sox30 was implicated in “Phenylalanine metabolism” (mmu00360) and “Glycolysis/Gluconeogenesis” (mmu00010) in spermatogonia, spermatocytes, spermatids, and Sertoli cells; Sox30 was involved in “Proteasome” (mmu03050) and “Nucleocytoplasmic transport” (mmu03013) in spermatocytes, spermatids and Sertoli cells; Sox30 was implicated in “Vasopressin-regulated water reabsorption” (mmu04962) and “Thermogenesis” (mmu04714) in spermatocytes, spermatids and Leydig cells, and “Oxidative phosphorylation” (mmu00190) in spermatocytes and spermatids. Sox30 was involved in “Ferroptosis” (mmu04216) in spermatocytes, Sertoli cells and Leydig cells, and was implicated in “Tight junction” (mmu04530) in the spermatocytes, Sertoli cells and Leydig cells. Sox30 seemed to be uniquely implicated in “Tyrosine metabolism” (mmu00350), “2-Oxocarboxylic acid metabolism” (mmu01210), “Mucin type O-glycan biosynthesis” (mmu00512), “Wnt signalling pathway” (mmu04310), “Drug metabolism-cytochrome P450” (mmu00982), “p53 signalling pathway” (mmu04115) and “Biosynthesis of amino acids” (mmu01230) in spermatogonia; Sox30 appeared to be exclusively involved in the “Regulation of actin cytoskeleton” (mmu04810) and “Protein export” (mmu04923) in spermatocytes, and implicated in “Pyrimidine metabolism” (mmu00240) and “Ribosome” (mmu03060) in spermatids (Fig. 7C; Supplementary Table S7, Supplementary Fig. S5). In Sertoli cells, Sox30 was implicated in the signals seem to be similar to the spermatids, while the “Drug metabolism-other enzymes” (mmu00983), “PPAR signalling pathway” (mmu03320), “SNARE interactions in vesicular transport” (mmu04130) and “Glycine, serine and threonine metabolism” (mmu00260) were probably unique in Sertoli cells; Except for “Ferroptosis” (mmu04216), Sox30 appeared to be exclusively involved in the “Vascular smooth muscle contraction” (mmu04270), “Viral protein interaction with cytokine and cytokine receptor” (mmu04061), “TGF-beta signalling pathway” (mmu04350), “cGMP-PKG signalling pathway” (mmu04022), “Cellular senescence” (mmu04218) and “Thyroid hormone synthesis” (mmu04918) in Leydig cells (Fig. 7C; Supplementary Table S7, Supplementary Fig. S5).

Taken together, these findings indicate that primary roles of Sox30 on testicular cells involve in spermatid development and differentiation. In addition, Sox30 was probably involved in the cell motility of testicular germ cells and somatic cells by influencing cilium events. However, Sox30 may regulate the features of different testicular cells via diverse molecular pathways. Sox30 may regulate spermatogonia preferentially through tyrosine metabolism, the Wnt signalling pathway and the p53 signalling pathway; regulate spermatocytes preferentially through influence actin cytoskeleton regulation and protein export; regulate spermatids preferentially via pyrimidine metabolism and ribosome regulation; regulate Sertoli cells preferentially by PPAR signalling pathway, SNARE interactions in vesicular transport, glycine, serine and threonine metabolism; and regulate Leydig cells preferentially via cGMP-PKG signalling pathway, TGF-beta signalling pathway, thyroid hormone synthesis and cellular senescence. The discrepancy in the enrichment of terms and pathways implies the diverse functions and mechanisms of Sox30 in different testicular cells.

## Discussion

In this study, we determined the functional landscape and precise role of Sox30 in male germ cell development and differentiation via using scRNA-seq of testes from Sox30-null mice. Sox30 plays important roles in different testicular cell types and predominantly controls the meiosis and differentiation of spermatocytes, which further corroborates our previous findings [29]. The impairments of Sox30-null on different testicular cells possesses clear functional and mechanistic characteristics. This study highlights the exhaustive functional and mechanistic atlas and features of Sox30 in testicular cells.

According to the expression of markers [9, 13, 42], ten distinct cell types including spermatogonia, spermatocytes, spermatids, endotheliocytes, macrophages, T cells, SMCs, Sertoli cells, Leydig cells and unknown cells, were identified via scRNA-seq. Sox30 deficiency significantly alters the constituent ratio of testicular cells, resulting in a clear decrease in spermatids and an increase in spermatocytes, spermatogonia, unknown cells, Sertoli cells, Leydig cells, SMCs, macrophages and T cells. Pathological analyses strongly confirmed the findings of the scRNA-seq. These results imply that Sox30 is likely to influence various types of testicular germ cells and somatic cells. Moreover, multi-nucleated germ cells were found in the seminiferous tubules of Sox30^−/−^ testes as previously reported [27, 29, 30]. Consistently, the unknown cell cluster accumulated in Sox30^−/−^ testes in the present study, and they highly expressed marker genes for spermatocytes and spermatids, and shared the activated TFs of these two cell types, suggesting that the unknown cell cluster should correspond to the multi-nucleated cells observed in pathology. The reason for the appearance of this multi-nucleated cells may be the abnormal division, i.e., abnormal mitosis or meiosis, of germ cells due to Sox30 deficiency. As the multi-nucleated cells possess the properties of both spermatocytes and spermatids, both of which are deprived of mitotic activity, the reasonable explanation is the abnormality of germ cell meiosis due to Sox30 deletion.

Focusing on the exact roles of Sox30 in major testicular cells, distinct subclusters of germ cells and somatic cells were further identified, and the germ cells seemed to be largely arrested at the early stage of meiosis I, from leptotene to metaphase spermatocytes, in Sox30-null mice. A significant increase in the proportion of primary spermatocytes was observed from both pathological and scRNA-seq data, and the accumulation of spermatocytes at the zygotene stage, as we previously reported [29], was also observed in Sox30-null mice. Successfully differentiated secondary spermatocytes appeared to be absent, which may be the underlying cause of the dramatic reduction in the number of spermatids in Sox30-null mice. Sox30 seems to affect all of the major testicular cells, and among these cells, the most affected cell type appears to be the meiotic spermatocytes. To further confirm these results, the developmental trajectory of the major testicular cells was determined. Sox30 primarily controls the developmental and differentiated fates of spermatocytes, but slightly influences those of other germ cells and somatic cells, which is clearly consistent with the major role of Sox30 in the meiotic spermatocytes. Sox30 has been reported to be highly expressed in spermatocytes and subordinately expressed in round spermatids [29, 32], suggesting that important gene program regulated by Sox30 is more likely to occur first in spermatocytes. Spermatocytes appear to be nearly arrested at meiosis I in Sox30^−/−^ mice, and the expression of a panel of key meiotic regulators has been found to be regulated by Sox30 [29, 31]. These data strongly indicate the decisive role of Sox30 in determining the developmental and differentiation fates of spermatocytes. Unexpectedly, an increase in the proportion of type A spermatogonia is observed in Sox30^−/−^ mice, revealing potential roles for Sox30 in regulating spermatogonial proliferation and/or differentiation. These results highlight the indispensable role of Sox30 in initiating and promoting meiosis in spermatocytes. Moreover, the effects of Sox30 on male germ cell development and differentiation even begin in spermatogonia, and further studies are needed to clarify this issue.

The distinct subclusters of somatic Sertoli cells and Leydig cells were also further identified and analysed. The proportions of immature-like Sertoli cells and mature-like Leydig cells clearly increased in Sox30-null mice, suggesting that Sox30 plays important roles in regulating mature phenotypes of Sertoli cells and Leydig cells. Given the effects of Sox30 on mature of Sertoli cells and Leydig cells, Sox30 can undoubtedly regulate the levels of hormones secreted by these cells. Notably, the number of isolated Leydig cells is limited, which may introduce some inaccuracy in our present data. In fact, our annotation results refer to many previously reported Leydig cell markers [58–62] and we distinguish these cell subclusters on the basis of their main functional characteristics (including sterol synthesis ability and IGF expression), which could reflect the maturation of Leydig cell populations to a certain extent. Indeed, the levels of retinoic acid, inhibin B and testosterone produced by Sertoli cells and Leydig cells are dysregulated in Sox30-null mice [29, 64], which further confirms the conclusion that Sox30 regulates the maturation of Sertoli and Leydig cells.

The effects of Sox30 on the complex network of interactions among major testicular cells were further determined. The communication of spermatogonia–Leydig cell, spermatocyte–Leydig cell, spermatid–Leydig cell and Sertoli cell-Leydig cell was significantly dysregulated in Sox30-null mice. Furthermore, dysregulated communication was also observed between subclusters of germ cells and somatic cells in Sox30-null mice. The alterations in cross-talk among germ cell and somatic cell (sub)clusters probably contribute to the destruction of the spermatogenic microenvironment and eventually dyszoospermia. Leydig cells drive spermatogenesis via the secretion of testosterone. Some studies have shown that testosterone does not act directly on germ cells but rather acts on Sertoli and/or peritubular cells to create an environment [65, 66]. Leydig cells may have complex interactions with germ cells, Sertoli cells and vasculature, and germ cell-derived signals can directly or indirectly affect Sertoli and Leydig cell populations during testis development [67]. In our results, the reduced interactions between germ cells and Leydig cells/Sertoli cells in Sox30 null mice at least suggest disruption of the environment created by Leydig cells and Sertoli cells. In fact, the receptor ligands and TFs displayed in Sankey plot of these cells are mainly involved in spermatogenesis, blood-testis barrier (BTB) homeostasis and testicular cell development. The altered interactions between spermatocytes and endothelial cells, as well as between spermatids and endothelial cells, may further imply the disruption of the BTB, given the lack of such signalling interactions in wild-type mice. Additionally, alterations in Sertoli cell functions, such as reduced levels of inhibin B in Sox30^−/−^ mice [64], are consistent with disrupted cell-to-cell communication. These data suggest an important role of Sox30 in maintaining spermatogenesis and the spermatogenic microenvironment. TFs have been reported to be important hubs that regulate cell fate transition [68]. As a member of the TF, Sox30 itself involved in the transcription of multitudinous genes and influences the expression of other TFs. The results of the TF network analyses revealed that Sox30 affects mainly the TF network of spermatocytes, followed by that of Leydig cells, spermatogonia, spermatids and Sertoli cells. The altered activities of the TFs of these cells in Sox30^−/−^ mice are likely to be involved primarily in developmental processes, the meiotic cycle, and the cell cycle/differentiation/proliferation. In spermatogonia, Sox30 loss results in dysregulation of the TFs that regulate the cell cycle, development and differentiation. Similar functions of Sox30 are also observed in Sertoli cells and Leydig cells by regulating the activities of different TFs. Sox30 deletion in spermatocytes disrupts a batch of TF activities involved in the meiotic cycle and cell proliferation, which is consistent with the abnormal development and meiotic state of spermatocytes. Furthermore, we observed more changes in functional TF activities in different subclusters of testicular cells, and these altered TFs can indicate the cell status at different stages. The altered proportion of spermatogonia subclusters is probably related to the imbalance of TFs and their activities involved in the regulation of cell proliferation, differentiation and apoptosis. An imbalance of TFs involved in cell proliferation and differentiation (e.g., *Dmrt1*, *Foxa2*, and *Sox12*) is observed in spermatogonia_1 (type A spermatogonia). However, the regulon activities of TFs involved in DNA repair, cell division and even stemness maintenance (such as *Bach1*, *Kdm5a*, *Brca1*, and *Hinfp*) are significantly enriched in different spermatocyte clusters in Sox30-null mice. Only a few TFs are unaffected by Sox30 in spermatocyte_1 (pSPC), whereas the TFs activated in spermatocyte_3 (sSPC) seem to be almost not activated in the spermatocyte subclusters of Sox30-null mice. These data suggest that the meiosis of spermatocytes is arrested.

The major functions of Sox30 seem to closely resemble those of the major testicular cells according to enrichment analyses. Spermatogenic development and differentiation are preferentially enriched in germ cell, Sertoli cell and Leydig cell clusters, indicating the critical role of Sox30 in spermatogenesis, and the DEGs involved in these biological events constitute the group of genes responsible for meiotic transition and spermatid development. Alterations in cilium events involved in cell motility were also significantly enriched in the major testicular cells after Sox30 deletion. Ciliary assembly (ciliogenesis) and disassembly are dynamically regulated with cell cycle progression [69], so cilium events in spermatogonia and somatic cells may be associated with the cell mitotic cycle, which in turn regulates cell growth and division status. However, in male meiotic cells, cilia appear only briefly at the transition from leptotene to zygotene prior to mother centriole duplication in primary spermatocytes [47], and the meiotic cilium with cell signalling processes in this case is likely related to the initiation of synapsis at zygotene [70, 71]. Synaptonemal complex assembly and homologous chromosome pairing, which mainly occur during the zygotene stage, are the important events in the meiosis of spermatocytes [72]. In our previous study, the arrested stage of zygotene in spermatocytes seemed be attributed to the disruption of the synaptonemal complex in Sox30^−/−^ mice [29]. These findings suggest that Sox30 is likely involved in synaptonemal complex assembly and homologous chromosome pairing via regulation of cell cilium events.

According to the KEGG enrichment analyses, Sox30 appears to function in various testicular cells through different signalling pathways. In germ cells and Sertoli cells, phenylalanine metabolism and glycolysis/gluconeogenesis are significantly altered after Sox30 loss, suggesting that Sox30 may regulate amino acid and glucose metabolism in these testicular cells. In the testis, the Wnt signalling pathway specifically contributes to the proliferation of undifferentiated spermatogonia [73], whereas p53 signalling is related to the proliferation and apoptosis of spermatogonia [74, 75]. In our present study, Sox30 appeared to be uniquely involved in the Wnt signalling pathway and p53 signalling pathway in spermatogonia, indicating that the effect of Sox30 on spermatogonia proliferation/differentiation is associated with the Wnt and p53 signalling pathways. Dynamic regulation of the actin cytoskeleton contributes to a range of cellular events, including cell migration, cell division, cell morphogenesis and autophagy [76, 77]. During spermatogenesis, the actin cytoskeleton not only provides structural support for cell morphology and movement but also participates in cell division, growth, differentiation, anchoring junctions and acrosome formation in germ cells [76, 78, 79]. In our present study, Sox30 seemed to specifically regulate the actin cytoskeleton and protein export in spermatocytes, and biological components such as acrosomal vesicles, sperm flagella and motile cilium were also significantly enriched in spermatocytes and spermatids. These data suggest that Sox30 may be involved in spermatocyte division and subsequent spermatid acrosome formation by specifically regulating the actin cytoskeleton of spermatocytes and even influencing cytoskeleton-dependent protein export. As well-known orchestrators of spermatogenesis, Sertoli cells commit to providing a hormonal environment, nutritional and structural support for the SSC niche, development and differentiation of spermatogonial populations, and meiosis [80]. Sox30-null uniquely altered SNARE interactions in vesicular transport, the PPAR signalling pathway, and glycine, serine and threonine metabolism in Sertoli cells. SNARE proteins present on all organelles are involved in intracellular vesicle trafficking and secretion [81], and SNARE deficiency may affect the normal membrane trafficking of Sertoli cells and impair the developmental ability of germ cells [82]. The PPAR protein and its receptors in Sertoli cells are thought to mediate glucose and fat metabolism to ensure energy metabolism in these cells and in germ cells [83, 84]. On the basis of the above descriptions, Sox30 is posited to influence energy metabolism and material transport in Sertoli cells through SNARE and the PPAR signalling pathway and may also affect the functions of Sertoli cells through the glycine–serine–threonine axis. Leydig cells are the major androgen-producing cells and are responsible for establishing the endocrine environment [85]. In the present study, Sox30 may participate in Leydig cell function by TGF-beta signalling, cGMP-PKG signalling, glutathione metabolism, and thyroid hormone synthesis. Interestingly, cellular senescence signalling was specifically observed in Leydig cells after Sox30-null, suggesting that Sox30 may preferentially regulate Leydig cell senescence in testicular cells. In addition, ferroptosis appears to be preferentially enriched in spermatocytes, Sertoli cells and Leydig cells, indicating that Sox30 may preferentially participate in the ferroptosis of spermatocytes, Sertoli cells and Leydig cells. However, whether Sox30 is involved in iron homeostasis through specific signalling pathways such as glutathione metabolism, in these cells, is worth further exploration.

A question may be raised that it is difficult to understand the role of Sox30 deletion in testicular cells that physiologically do not express Sox30. The reasonable explanation is that Sox30 may directly act roles on these testicular cells (spermatogonia, Leydig and Sertoli cells) since observable expression has been reported, or that abnormalities in the spermatogenic cycle caused by Sox30 acting on spermatocytes and round spermatids likely provide functional feedback to other testicular cells. (1) Previous studies and our data revealed that the high expression of Sox30 is restricted to spermatocytes and round spermatids [27, 29, 31]. However, Sox30 was observed to be expressed in spermatogonia, Leydig cells and Sertoli cells [24, 29, 32]. (2) Sox30, a transcription factor (TF), plays an important role in controlling cell fate transition. Our data revealed, significant alterations in the TF networks in both germ cells and somatic cells in Sox30-null mice, and different cell types and subtype populations can be distinguished by the different altered TFs. Thus, the misregulation of TF networks in testicular cells from Sox30 deletion mice may alter the development of other testicular cells. (3) Intercellular communication contributes to regulation and maintenance of the testicular niche and germ cell development [5]. In our present study, the ligand/receptor signals were strongly altered among germ cells and somatic cells, implying that Sox30 deletion significantly alters the communication between different testicular cells. The destruction of the cross-talk of spermatocytes and round spermatids with other testicular cells in Sox30 deletion mice likely affect the development of other testicular cells. To determine the exact functions of Sox30 in all major testicular cells, the construction of a conditional knockout mouse model in each specific testicular cell type is necessary to evaluate the differences in Sox30 function. We are working hard on this, but it would take a long time and tedious attempts.

**Fig. 8:**
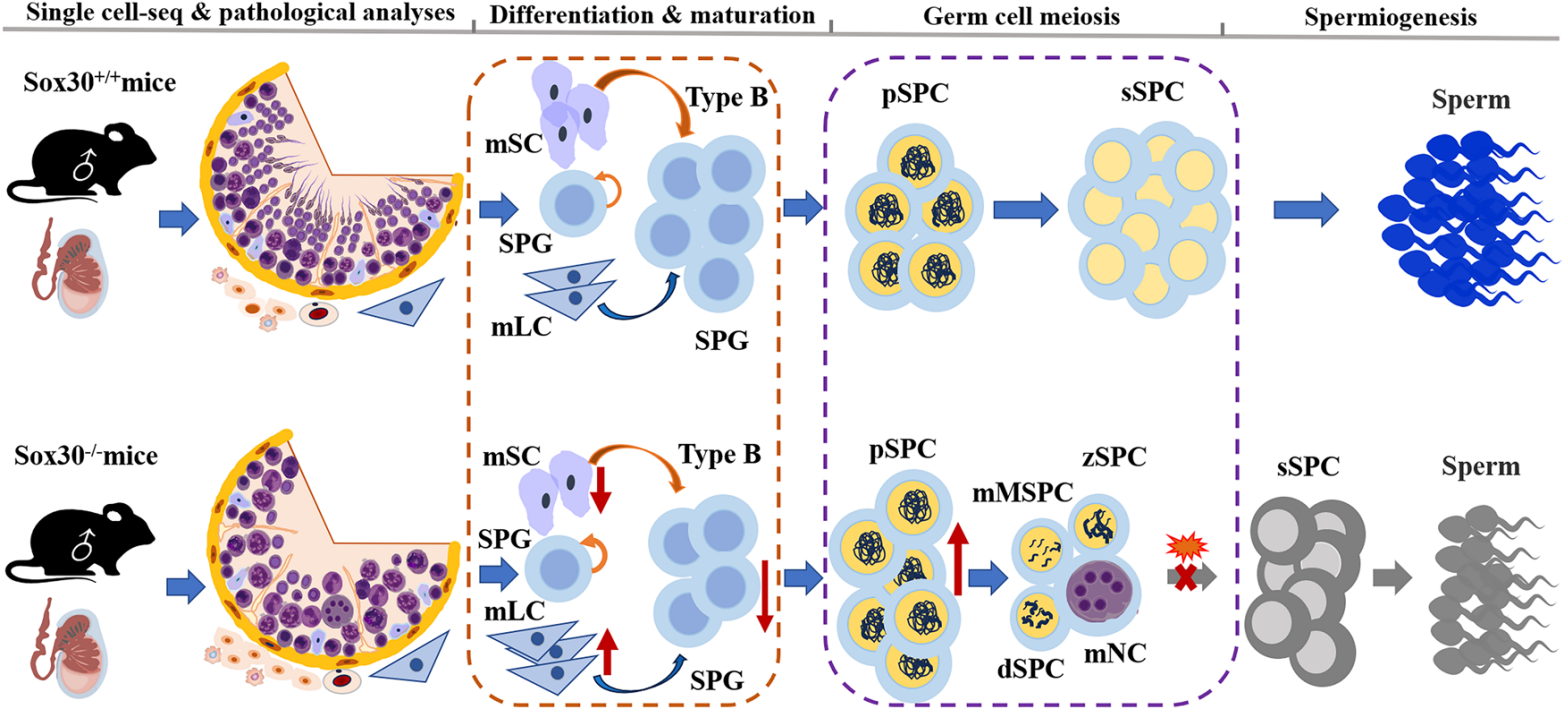
Schematic illustration for the precise effects of Sox30 on major testicular cells. Sox30 primarily drives the development and differentiation fate of spermatocytes. Sox30-null results in spermatocytes being arrested at meiosis I, leading to azoospermia. At the same time, Sox30 likely also plays important roles in regulating the proliferation and differentiation of spermatogonia, as well as the mature phenotypes of Sertoli cells and Leydig cells. The red arrows represent the upregulation or downregulation of proportions for testicular cells. The SPG, type B, pSPC, sSPC, mSC, mLC, mMSPC, zSPC, dSPC, mNC represent spermatogonia, type B spermatogonia, primary spermatocytes, second spermatocytes, maturate Sertoli cells, mature Leydig cells, meiosis I metaphase spermatocytes, zygotene-like spermatocytes, diplotene-like spermatocytes, and multi-nucleated cells, respectively.

In summary, Sox30 primarily drives the development and differentiation spermatocytes, and loss of *Sox30* results in spermatocytes arrested at meiosis I, which is probably the rationale cause of azoospermia. Moreover, Sox30 also plays important roles in regulating the proliferation and differentiation of spermatogonia and mature phenotypes of Sertoli cells and Leydig cells (Fig. 8). Sox30 plays the key roles by regulating the developmental trajectories, intercellular cross-talk, and transcription factor networks of these major testicular cells. Mechanistically, Sox30 seems to have similar functions in all major testicular cells, whereas these similar functions may be mediated by different signalling pathways in different testicular cells. The present study defines and extends the crucial roles of Sox30 in testicular cell development and differentiation. The revelation of Sox30 properties in male testis provides enticing possibilities of its role in fine meiotic process and far-ranging spermatogenesis, testicular cell homeostasis regulation and additional male infertility.

## Supplementary information

Supplementary Figures and table legends

Supplemental Table S1

Supplemental Table S2

Supplemental Table S3

Supplemental Table S4

Supplemental Table S5

Supplemental Table S6

Supplemental Table S7

## Data Availability

All the data are available in the main text and supplemental materials, and the raw data can be obtained by contacting the corresponding author.
