# Supplementary material for: Single-cell RNA-seq and pathological phenotype reveal the functional atlas and precise roles of Sox30 in testicular cell development and differentiation
Source: Cell Death Dis. 2025 Feb 19;16(1):110. doi: 10.1038/s41419-025-07442-1 (PMC11840104; doi:10.1038/s41419-025-07442-1)
Supplement: Supplementary file 1 — Supplementary Figures and table legends [file 41419_2025_7442_MOESM1_ESM.pdf]

Supplementary figure and table legends

Supplementary figures and figure legends

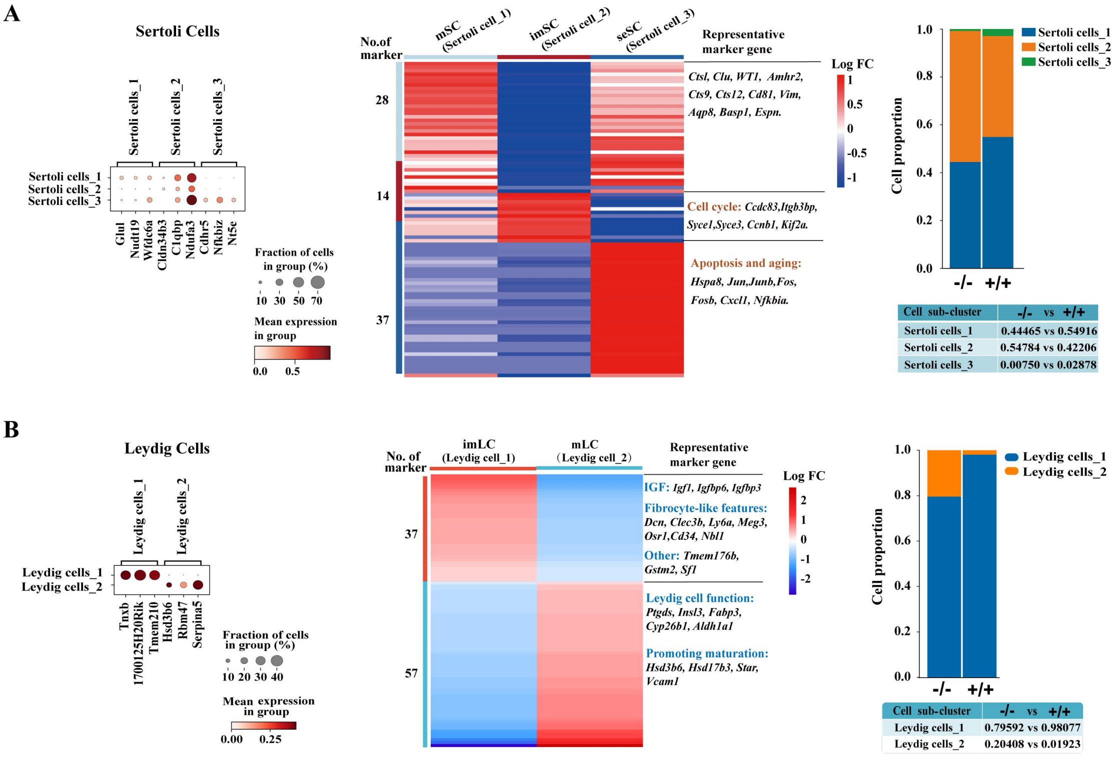

Figure S1. The identification and cell proportion of sub-clusters in the major testicular cells

(A-B) From left to right: Bubble chart for expression of 3 marker genes for per sub-cluster. Heatmaps showing the expression of representative marker genes of sertoli cells (A) and leydig cell (B) for each sub-cluster. Bar graphs and table representing the sub-cell proportions in Sox30$^{+/+}$ (+/+) and Sox30$^{-/-}$ (-/-) mice.

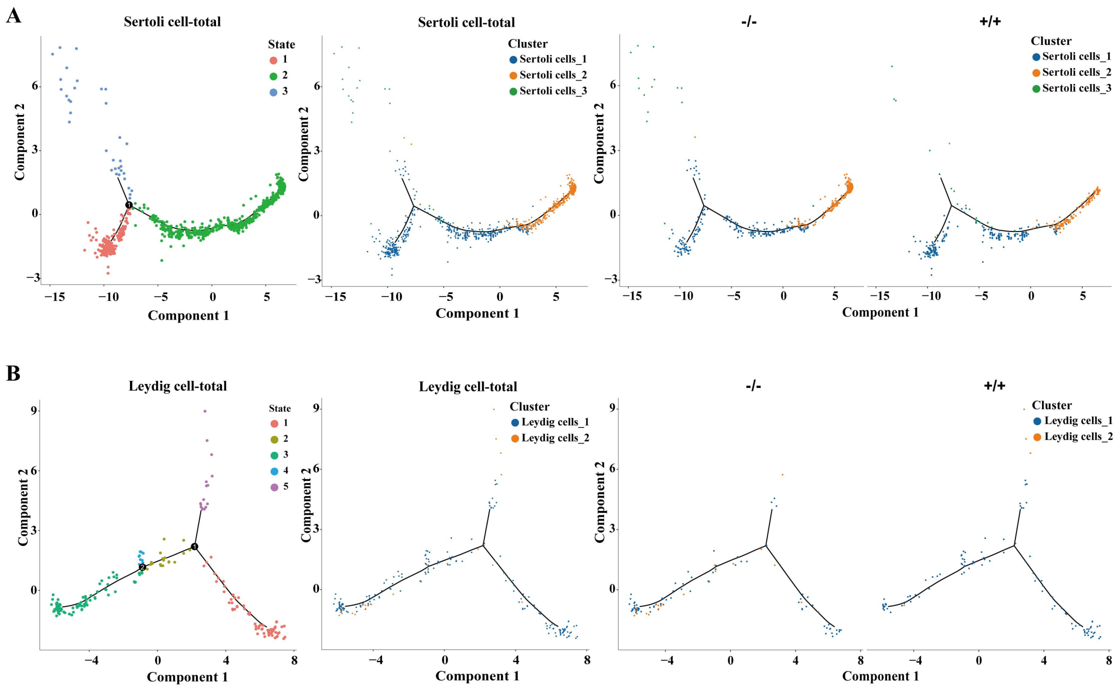

Figure S2. The development trajectories of major testicular somatic cells in Sox30$^{+/+}$ and Sox30$^{-/-}$ mice

(A) From left to right: the cell trajectories of integrated sertoli cells ordered in cell state (left) and integrated sertoli cells (middle), Sox30$^{-/-}$ (-/-) sertoli cells, Sox30$^{+/+}$ (+/+) sertoli cells colored according to UAMP cell clusters (right). (B) From left to right: the cell trajectories of integrated leydig cells ordered in cell state (left) and integrated leydig cells (middle), Sox30$^{-/-}$ (-/-) leydig cells, Sox30$^{+/+}$ (+/+) leydig cells colored according to UAMP cell clusters (right).

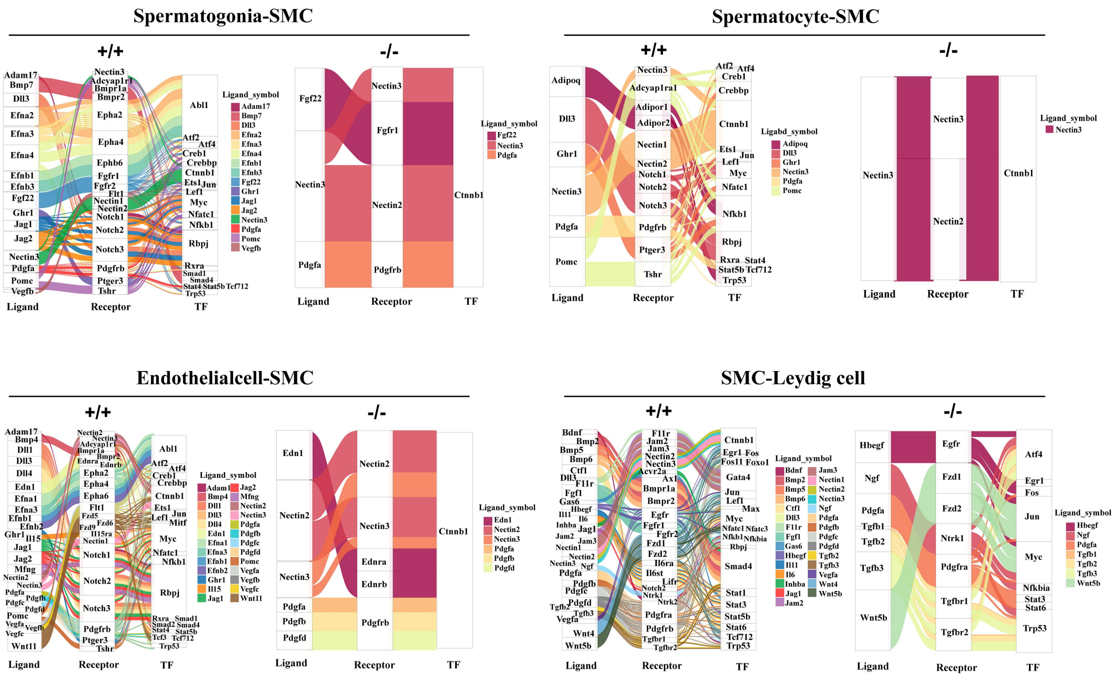

Figure S3. Sankey plot of ligand-receptor and interaction network of crucial TFs

Sankey plot of ligand-receptor and interaction network of crucial TFs among spermatogonia-SMC, spermatocyte-SMC, endothelial cell-SMC, and SMC-leydig cell in Sox30$^{+/+}$ (+/+) and Sox30$^{-/-}$ (-/-) mice, respectively. Each ligand assigned to different color, and line width represents strength of interaction.

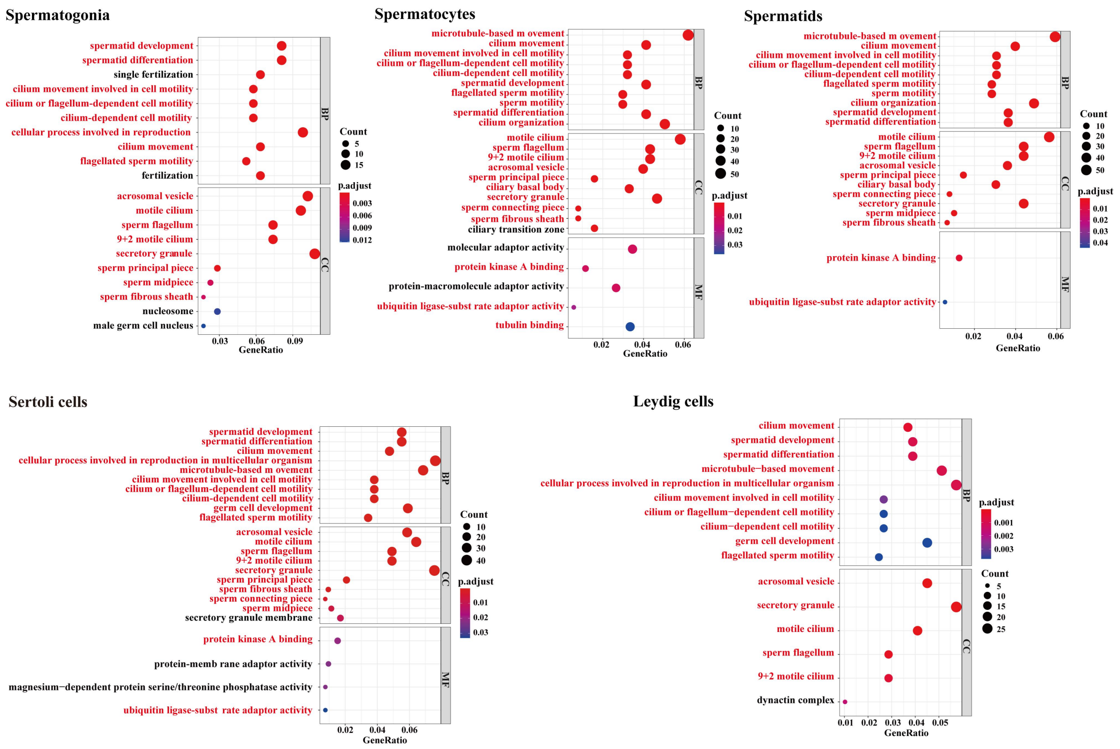

Figure S4. The possible key functions of Sox30 on major testicular cells

Bubble plot showing the top 10 GO terms for spermatogonia, spermatocyte, spermatid, sertoli cell and leydig cell clusters using the differentially expressed genes (DEGs) identified in each testicular cell cluster in Sox30$^{-/-}$ mice. The GO terms that are overlapped in different cell clusters are marked in red.

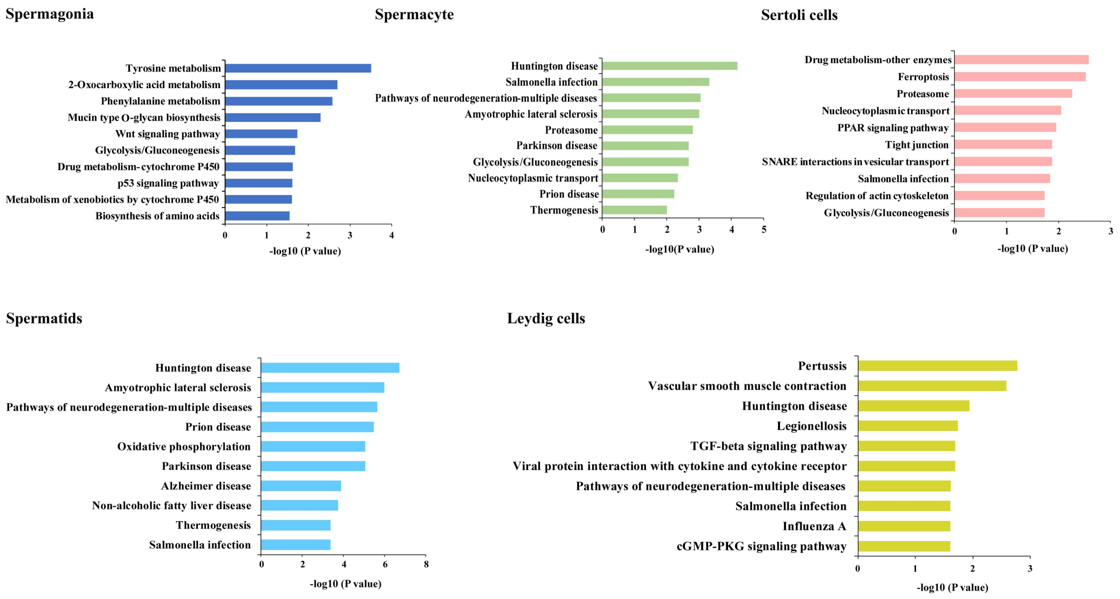

Figure S5. The potential pathways involved in the roles of Sox30 on major testicular cells

Bar chart showing the top 10 KEGG pathways (ranked by p-value) for spermatogonia, spermatocyte, spermatid, sertoli cell and leydig cell clusters using the differentially expressed genes (DEGs) identified in each testicular cell cluster in Sox30$^{-/-}$ mice.

Supplementary table legends

Supplementary Table S1. The marker genes of cell clusters.

Supplementary Table S2. The marker gene of sub-cell clusters.

Supplementary Table S3. The markers for identification of different stages testicular cells.

Supplementary Table S4. Pathological data of positive cell number in seminiferous tubule.

Supplementary Table S5. The co-expression and uniqueness TFs in $Sox30^{+/+}$ and $Sox30^{-/-}$ mice.

Supplementary Table S6. The GO enrichments of different testicular cells.

Supplementary Table S7. The KEGG enrichments of different testicular cells.
